# Preclinical Evaluation of [^18^F]FACH in Healthy Mice and Piglets: An ^18^F-Labeled Ligand for Imaging of Monocarboxylate Transporters with PET

**DOI:** 10.3390/ijms22041645

**Published:** 2021-02-06

**Authors:** Daniel Gündel, Masoud Sadeghzadeh, Winnie Deuther-Conrad, Barbara Wenzel, Paul Cumming, Magali Toussaint, Friedrich-Alexander Ludwig, Rareş-Petru Moldovan, Mathias Kranz, Rodrigo Teodoro, Bernhard Sattler, Osama Sabri, Peter Brust

**Affiliations:** 1 Helmholtz-Zentrum Dresden-Rossendorf, Institute of Radiopharmaceutical Cancer Research, Department of Neuroradiopharmaceuticals, Research Site Leipzig, 04308 Leipzig, Saxony, Germany; m.sadeghzadeh@hzdr.de (M.S.); w.deuther-conrad@hzdr.de (W.D.-C.); b.wenzel@hzdr.de (B.W.); m.toussaint@hzdr.de (M.T.); f.ludwig@hzdr.de (F.-A.L.); r.moldovan@hzdr.de (R.-P.M.); Mathias.Kranz@unn.no (M.K.); r.teodoro@hzdr.de (R.T.); p.brust@hzdr.de (P.B.); 2 Department of Nuclear Medicine, Inselspital, Bern University, 3010 Bern, Switzerland; paul.k.cumming@gmail.com; 3 School of Psychology and Counselling, Queensland University of Technology, Brisbane 4000, Australia; 4 Department for Nuclear Medicine, University Hospital Leipzig, 04103 Leipzig, Saxony, Germany; bernhard.sattler@medizin.uni-leipzig.de (B.S.); osama.sabri@medizin.uni-leipzig.de (O.S.)

**Keywords:** monocarboxylate transporters (MCTs), [^18^F]FACH, PET imaging, cancer, metabolism, kidney

## Abstract

The expression of monocarboxylate transporters (MCTs) is linked to pathophysiological changes in diseases, including cancer, such that MCTs could potentially serve as diagnostic markers or therapeutic targets. We recently developed [^18^F]FACH as a radiotracer for non-invasive molecular imaging of MCTs by positron emission tomography (PET). The aim of this study was to evaluate further the specificity, metabolic stability, and pharmacokinetics of [^18^F]FACH in healthy mice and piglets. We measured the [^18^F]FACH plasma protein binding fractions in mice and piglets and the specific binding in cryosections of murine kidney and lung. The biodistribution of [^18^F]FACH was evaluated by tissue sampling ex vivo and by dynamic PET/MRI in vivo, with and without pre-treatment by the MCT inhibitor α-CCA-Na or the reference compound, FACH-Na. Additionally, we performed compartmental modelling of the PET signal in kidney cortex and liver. Saturation binding studies in kidney cortex cryosections indicated a *K_D_* of 118 ± 12 nM and *B_max_* of 6.0 pmol/mg wet weight. The specificity of [^18^F]FACH uptake in the kidney cortex was confirmed in vivo by reductions in AUC_0–60min_ after pre-treatment with α-CCA-Na in mice (−47%) and in piglets (−66%). [^18^F]FACH was metabolically stable in mouse, but polar radio-metabolites were present in plasma and tissues of piglets. The [^18^F]FACH binding potential (BP_ND_) in the kidney cortex was approximately 1.3 in mice. The MCT1 specificity of [^18^F]FACH uptake was confirmed by displacement studies in 4T1 cells. [^18^F]FACH has suitable properties for the detection of the MCTs in kidney, and thus has potential as a molecular imaging tool for MCT-related pathologies, which should next be assessed in relevant disease models.

## 1. Introduction

Monocarboxylates such as L-lactate, pyruvate, and ketone bodies are important substrates for energy metabolism in living tissues. Being negatively charged at physiological pH, monocarboxylic acids do not diffuse readily across plasma membranes, but are trafficked by facilitated diffusion mediated by a family of monocarboxylate transporters belonging to the superfamily of the solute carrier transporters (SLC16). Among the numerous MCT types, MCT1-4 are the most thoroughly characterized in mammals with respect to their expression patterns and substrate specificities. They are passive transporters driven by proton and substrate gradients across plasma membranes, thus leading to substrate influx or efflux. In contrast to MCT1, the expression of MCT4 is controlled by HIF-1-alpha and is therefore overexpressed by hypoxic cells to increase their export of lactate produced by glycolysis [1,2,3,4,5]. 

The human body produces about 1.5 moles of lactate each day, mainly in skeletal muscle, skin, brain and erythrocytes [6,7]. Since this corresponds to roughly 500 kcal per day, it is unsurprising that there is considerable lactate uptake and metabolic salvage by the Cori cycle (gluconeogenesis), mainly occurring in liver and kidney [8]. Glomerular filtration of lactate in the human kidney is followed by 95% reabsorption by sodium-coupled monocarboxylate transporters 1 and 2 (SMCT1/2) expressed at the apical side of the proximal tubules, which is then directed either to gluconeogenesis or recirculated back to blood via MCT1 [2,9,10]. Indeed, kidney expresses several different monocarboxylate transporters, although only MCT1 and MCT8 (which is specific for thyroid hormones) are expressed at the basolateral site of endothelial cells in the S1/S2 parts of the convoluted proximal tubules in renal cortex [11,12].

MCT expression is subject to metabolic adaption, for example in the skeletal muscle, where lactate uptake driven by MCT1 expression is potentiated by long term physical conditioning [13], or in the liver, where the expression of MCT1 and MCT2 increases in starved mice [14]. Changes in the MCT1 and MCT4 expression are also linked to diverse patho-physiologies, including myocardial ischemia [15], cancer [3,16], acidosis [6,17], hyperinsulinemia [18], diverse liver [19] and inflammatory bowel diseases [20], as well as brain diseases such as ischemic brain injury, epilepsy, and neurodegenerative conditions [21,22]. Hence, MCT expression and lactate shuttling change along with altered metabolic requirements of tissues.

**Figure 1:**
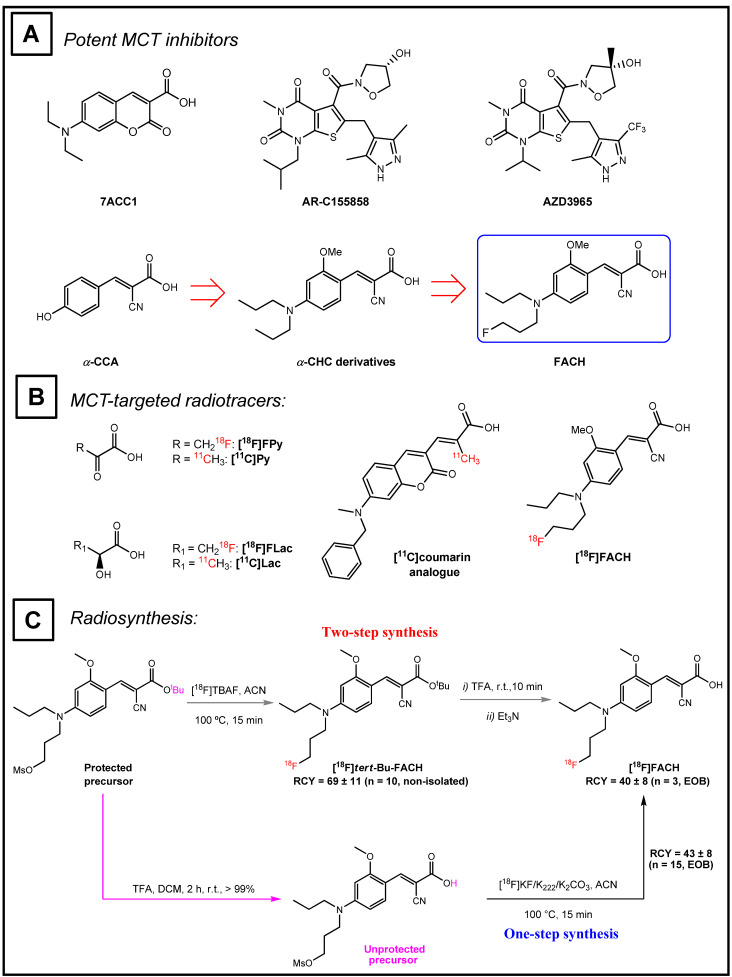
(**A**) Some potent MCT inhibitors and schematic pathway showing the development of FACH. (**B**) Representative ^18^F- or ^11^C-labeled MCT substrates and inhibitors. (**C**) Two-step one-pot vs. one-step procedure for radiosynthesis of [^18^F]FACH [28,29].

MCT expression could potentially serve as clinical marker for better patient stratification and individualized treatment adaptions. For example, altered expression of MCT1 and MCT4 has been reported in many tumour types, including gliomas, breast cancer, carcinoma of renal cells, colorectal cancer and squamous tissues, as well as cervical and lung cancers [3]. Therefore, several potent MCT1/4 inhibitors have been developed for potential cancer treatment (Figure 1A). Additionally, sensitive and specific probes for molecular imaging of the MCT1/4 could prove invaluable tools for clinical research. In this context, ^11^C- and ^18^F-labeled substrates and inhibitors of the MCTs have emerged as molecular imaging agents for use in positron emission tomography (Figure 1B). For instance, (±)-[^18^F]-3-fluoro-2-hydroxypropionate was developed for imaging MCT-dependent lactate uptake in cancer cells, whereas a ^11^C-labeled coumarin analogue was developed for detecting MCT expression [23,24]. Furthermore, lactate metabolism has been imaged with *L*-3-[^11^C]lactate in heart [25], and pyruvate turnover measured in brain with 1-[^11^C]pyruvate [26] and in tumours with [^18^F]fluoropyruvate [27]. Recently, our group developed a novel PET radiotracer, (E)-2-cyano-3-{4-[(3-[^18^F]fluoropropyl)(propyl)amino]-2-methoxyphenyl}acrylic acid ([^18^F]FACH) [28,29], which is a highly potent analogue of the known MCT inhibitor α-cyano-4-hydroxycinnamic acid (α-CCA), with a ten-fold higher selectivity towards MCT1/4 than other subtypes [30]. [^18^F]FACH has an IC_50_ value of 11.0 nM towards MCT1 and 6.5 nM towards MCT4 in *L*-[^14^C]lactate uptake assays, and an IC_50_ of 78 µM towards SMCT1 in a [^14^C]nicotinate uptake assay [28]. Radiosynthesis of [^18^F]FACH was already performed via two approaches which are shown in Figure 1C [28,29]. Furthermore, initial dosimetry studies in piglets predicted an effective radiation dose for humans of about 2 mSv/100 MBq [^18^F]FACH, which is acceptable for use in clinical research [31]. In the current study, we focused on the preclinical evaluation of [^18^F]FACH metabolism in vivo, its binding specificity and biodistribution in healthy mice and piglets. To estimate the binding potential (BP_ND_) of [^18^F]FACH in kidney and liver, we performed compartmental modelling of the acquired dynamic imaging data. Our results confirm the potential of [^18^F]FACH as a PET tracer for detecting disease-related changes in the MCT expression and function. Additionally, toxicology studies in rat were performed to determine the no-observed-adverse-effect-level (NOAEL) for human application.

## 2. Results

### 2.1. Plasma Protein Binding

The mean values of the free plasma fraction (*f*_p_) of [^18^F]FACH were 0.07 in mouse plasma (ex vivo) and 0.03 in pig plasma (in vitro).

### 2.2. In Vitro Determination of Binding Parameters of [^18^F]FACH in Mouse Tissues

**Figure 2:**
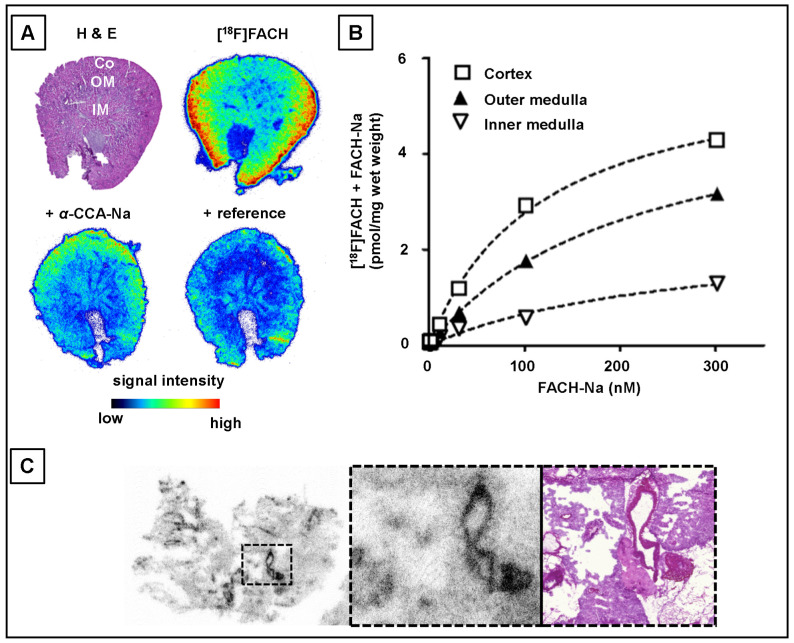
Binding studies of [^18^F]FACH on murine kidney (**A**,**B**) and lung (**C**) Cryosections incubated with 1 nM [^18^F]FACH. (**A**) Representative autoradiography of kidney (left) indicates a heterogeneous distribution of binding sites which resembles the gross-regions: cortex (Co), outer medulla (OM) and inner medulla (IM), detectable by H&E staining of the transversal tissue slice and signal intensity was reduced by competition with 300 nM α-CCA-Na or reference compound (FACH-Na). (**B**) Binding parameters for Co, OM and IM were estimated by non-linear regression analysis of saturation curves derived from a homologous displacement experiment using the reference compound and [^18^F]FACH, reflects similar affinities (*K_D_*: 118 nM, 212 nM, and 265 nM, resp.) but different densities (*B_max_*: 6.0, 5.4, and 2.4 pmol/mg wet tissue, resp.) of the binding sites. (**C**) Autoradiography of murine lung showing heterogenous binding sites of [^18^F]FACH (left), with a higher density in vascular structures (middle, dotted square from left magnified) and corresponding H&E staining (right).

Autoradiographic analysis of transverse sections of mouse kidney showed clearly delineated binding of [^18^F]FACH in three distinct sub-regions: cortex (Co), outer medulla (OM), and inner medulla (IM) (Figure 2A,B), with *B_max_* values of 6.0, 5.4 and 2.4 pmol/mg wet weight and corresponding *K_D_* values of 118 nM, 212 nM, and 265 nM, respectively. The autoradiographic competition study performed by co-incubation of [^18^F]FACH with 300 nM of the respective reference compound (FACH-Na) confirmed its higher affinity in comparison to 300 nM α-CCA-Na with 41% vs. 8% displacement of [^18^F]FACH (Figure 2A).

The autoradiographic distribution of the [^18^F]FACH binding in the mouse lung sections was heterogenous, with relatively higher concentrations of binding sites in blood vessels (Figure 2C).

### 2.3. Biodistribution of [^18^F]FACH in Mice

The biodistribution of [^18^F]FACH at five, 15 and 30 min post injection (p.i.) in mice was determined ex vivo by gamma-counting of weighed tissue samples (Table 1). The standardized uptake value (SUV) in whole blood declined from 1.85 ± 0.29 at five min to 0.82 ± 0.05 at 30 min p.i., whereas the plasma SUV was 3.77 ± 0.57 at five min and 1.69 ± 0.17 at 30 min p.i. Pre-treatment with α-CCA-Na led to significantly higher SUV in the blood (0.95 ± 0.06, *p* = 0.021) and in the plasma (1.92 ± 0.07, *p* = 0.044) at 30 min.

A steady increase of the kidney SUV from 7.9 ± 0.36 at five min to 15.97 ± 1.07 at 30 min after [^18^F]FACH injection was observed, whereas pre-treatment with α-CCA-Na led to a significantly reduced SUV of 5.94 ± 1.01 (*p* < 0.001) at 30 min p.i. The SUV in the lung declined from 0.89 ± 0.12 at five min to 0.52 ± 0.02 at 30 min after [^18^F]FACH injection. The SUVs in the lung, heart, and bones at 30 min p.i. were significantly increased after pre-treatment with α-CCA-Na. There were no significant changes in SUV after pre-treatment with α-CCA-Na in other tissues.

### 2.4. PET Studies in Mice

**Figure 3:**
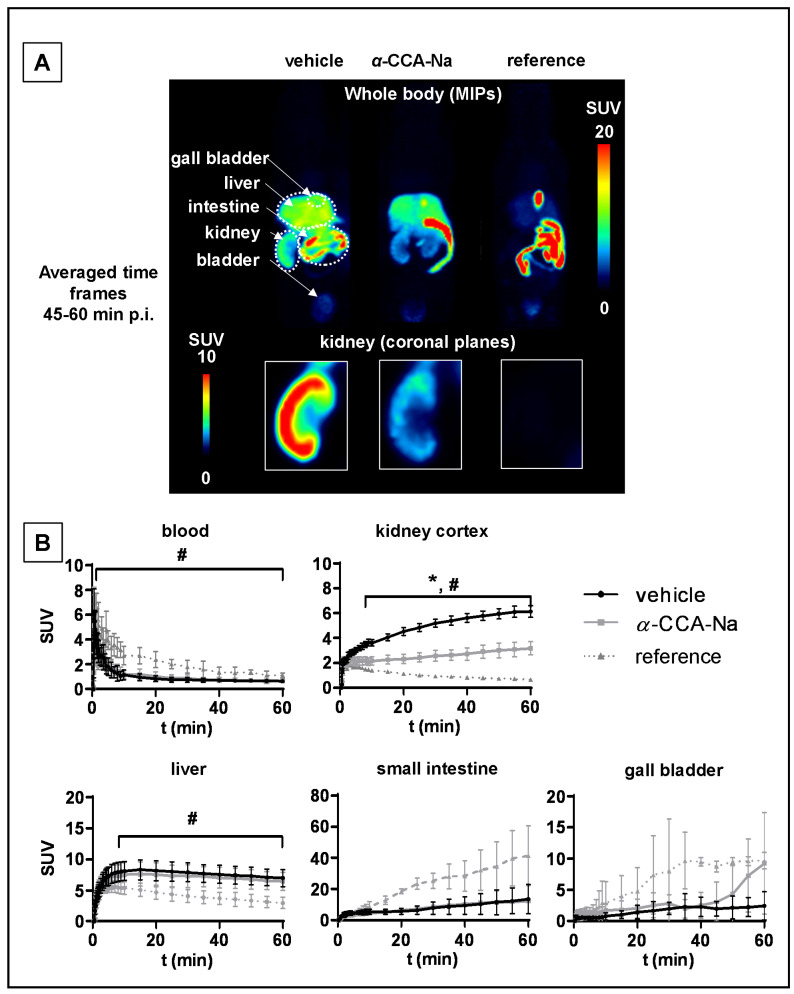
PET studies of [^18^F]FACH uptake in different tissues of mice pre-treated with vehicle (*n* = 10), 25 mg/kg bodyweight *α*-CCA-Na (*n* = 5), or 10 mg/kg reference compound (*n* = 3) at ten min before tracer injection. (**A**) Representative coronal whole body MIPs of PET images averaged from 45 to 60 min p.i. (**B**) Time-activity curves of blood, kidney cortex, liver, gall bladder and small intestine of mice, mean ± SD, *p* < 0.05 * *α*-CCA-Na and # reference compound pre-treated group vs. control group.

The values for the accumulated activity from 0–60 min (AUC_0–60min_) p.i. in mouse tissues are presented in Table 2. Dynamic PET images showed extensive uptake of [^18^F]FACH in the mouse kidney and other organs (Figure 3). The maximum intensity projections (MIPs) from the interval 45 to 60 min p.i. (Figure 3A) give a general overview of the [^18^F]FACH biodistribution and the MCT specific tissue uptake in mice by pre-treatment with α-CCA-Na and the reference compound, which clearly reduced radiotracer uptake in the kidney cortex.

The time activity curve (TAC) for the whole blood, derived from a volume of interest (VOI) placed in the left heart ventricle, indicated a peak TAC SUV of 6.8 ± 1.3 for all three treatment groups (Figure 3B). Significantly higher blood radioactivity concentrations were observed between 1.3 and 60 min p.i. in the group pre-treated with the reference compound, resulting in a significantly higher area under the curve from zero to 60 min (AUC_0–60min)_ compared to the control group.

Ex vivo studies of mouse tissues confirmed the specific renal uptake of [^18^F]FACH (Table 1), showing time-dependent increases of the uptake in the kidney cortex (Figure 3B), which is in accordance with the results of the binding studies in vitro (Figure 2). The uptake of [^18^F]FACH was significantly decreased in both pre-treated groups between eight and 60 min p.i. and resulted in a 47% lower AUC_0–60min_ upon pre-treatment with α-CCA-Na and 79% lower upon pre-treatment with the reference compound compared to control values (Figure 3B).

The [^18^F]FACH uptake in the liver of the control mice was considerably higher than that in the kidney. The pre-treatment with the reference compound reduced the liver uptake of [^18^F]FACH significantly between four and 60 min p.i. and led to a 44% reduction of the AUC_0–60min_. Interestingly, pre-treatment with α-CCA-Na had no significant impact on [^18^F]FACH uptake into the liver (Figure 3B). Additionally, contrary to effects of α-CCA-Na, pre-treatment with the reference compound increased the gall bladder uptake of [^18^F]FACH by 65% compared to the control mice. This apparently resulted in increased biliary excretion of the radioactivity and consequently elevated radioactivity accumulation in the intestine (Figure 3B).

**Figure 4:**
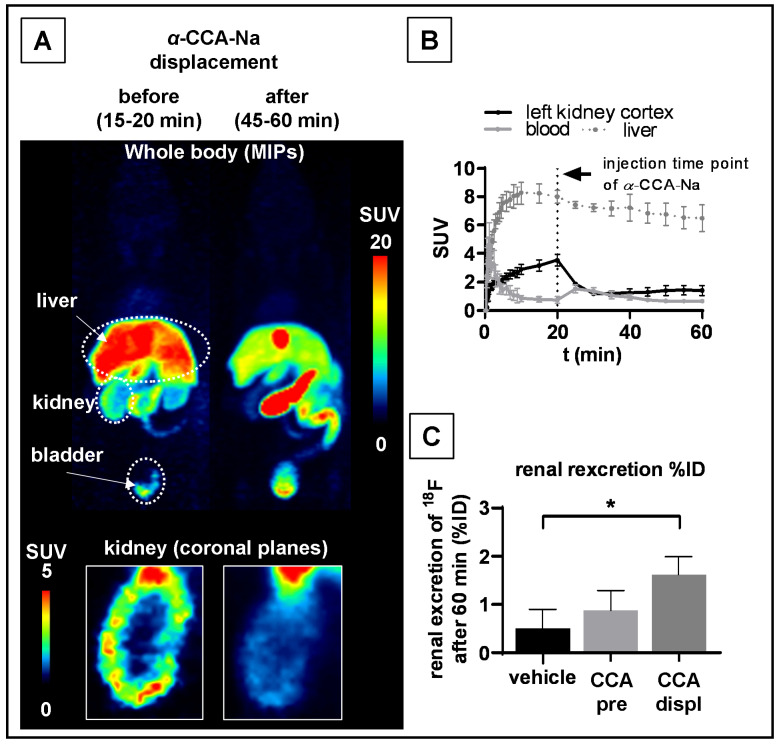
PET displacement study of [^18^F]FACH in mice treated with *α*-CCA-Na (25 mg/kg) at 20 min after tracer administration. (**A**) Representative whole body MIP of averaged SUVs from 15 to 20 min and from 45 to 60 min after tracer administration. (**B**) Time-activity curves of the kidney cortex, blood and liver with *α*-CCA-Na at 20 min after tracer application. (**C**) Renal excretion of radio-metabolites (CCA pre: *α*-CCA-Na injection ten min prior tracer administration; CCA displ: *α*-CCA-Na injection 20 min after tracer injection). Each point is the mean (±SD) of three determinations, *p* < 0.05 * treatment vs. control group.

Reversible binding of [^18^F]FACH in the mouse kidney cortex, but not in the liver, was confirmed by the displacement study in vivo, where α-CCA-Na was administered 20 min after the radiotracer administration (Figure 4A,B). The renal displacement was associated with a transient increase in the blood activity, which was elevated two-fold at five min after α-CCA-Na administration (SUV 1.50 ± 0.30 post- vs. SUV 0.74 ± 0.23 pre-injection of α-CCA-Na, *p* = 0.002). On the other hand, the kidney cortex SUV decreased by 66% from 3.50 ± 0.42 to 1.18 ± 0.09 five min after injection of α-CCA-Na and then slowly increased to 1.41 ± 0.36 towards the end of the 60 min recordings. There was no significant change in the liver, gall bladder and the intestine TACs upon displacement compared to the control studies (Figure S1). Interestingly, the displacement studies also showed significantly increased radioactivity in the mouse bladder at 60 min p.i. compared to the control group (1.6 ± 0.37 %ID vs. 0.51 ± 0.39 %ID) (Figure 4C).

### 2.5. PET Studies in Piglets

**Figure 5:**
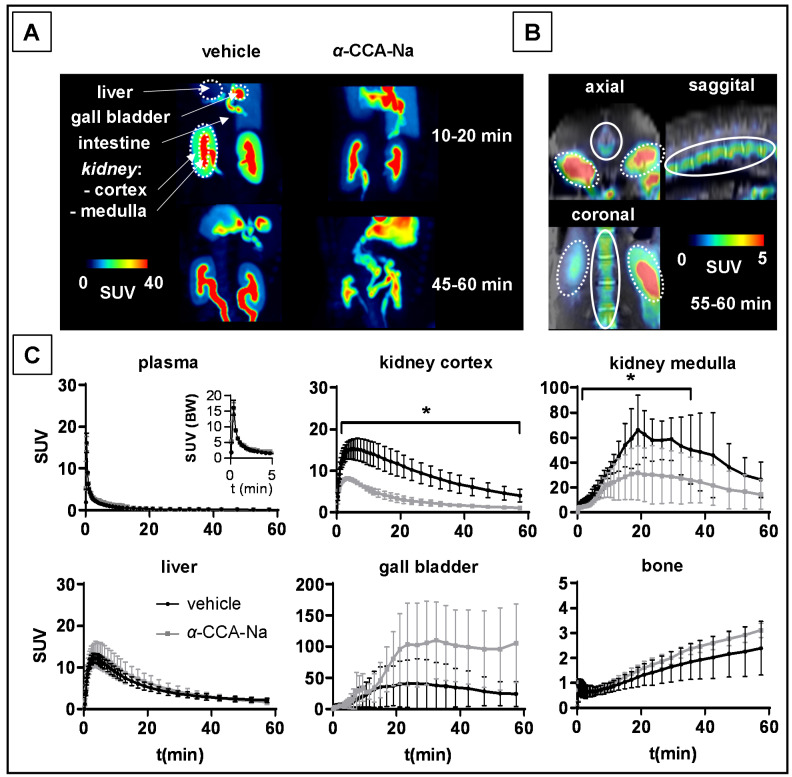
Dynamic PET results of [^18^F]FACH uptake in tissues of control piglets and in animals pre-treated with *α*-CCA-Na (25 mg/kg) at ten min prior to tracer administration. (**A**) Representative coronal abdominal MIPs at early (10 to 20 min) and late (45 to 60 min) phases of the PET recordings. (**B**) Representative axial, sagittal and coronal slices of averaged PET images with solid ellipse placed around the spine and dotted ellipses around the kidney. (**C**) Time activity curves of radio-metabolites and partial volume corrected, image-derived plasma input functions (where the insert depicts the first five min) and corresponding curves for the kidney cortex, kidney medulla, liver, gall bladder and bone (spine). Each point is the mean ± SD of three determinations, *p* < 0.05 * α-CCA-Na vs. control group.

The values for the accumulated activity from 0 to 60 min (AUC_0–60min_) p.i. in piglet tissues are presented in Table 3. The partial volume and radio-metabolite-corrected plasma input functions showed no significant difference in AUC_0–60min_ between the control and the α-CCA-Na pre-treated piglet groups. Contrary to findings in mice, there was no steady accumulation of the radioactivity over time in the kidney cortex of the control piglets. However, the peak TAC SUV in the kidney cortex (15.3 ± 2.5 at five min p.i.) was reduced by half in the α-CCA-Na pre-treated group (8.1 ± 0.4 at three min p.i.) as shown in Figure 5A,C and Figure S2. Accordingly, the AUC_0–60min_ dropped significantly by 66% in the kidney cortex. The peak TAC SUV at 19 min p.i. in the kidney medulla of the control piglets (66.1 ± 27.7) declined by half in the α-CCA-Na pre-treated group (31.6 ± 21.6) (Figure 5C). As well, significantly reduced SUVs were observed between 0.6 and 32.5 min p.i., which were accompanied by a 50% lower AUC_0–60min_ in the kidney medulla.

Regarding the lack of the pre-treatment effect by using α-CCA-Na on [^18^F]FACH uptake into the piglet liver (Figure 5C), we assume a non-specific component of radiotracer uptake in this tissue. The observed peak TAC SUV was 13.2 ± 2.7 in the control group vs. 13.3 ± 1.2 in the α-CCA-Na pre-treated animals, however, the corresponding AUC_0–60min_ values did not differ. Nevertheless, pre-treatment with α-CCA-Na increased the AUC_0–60min_ TACs in the gall bladder by two- to six-fold compared to the control group. The enhanced biliary excretion is in accordance with the results for kidney, which revealed an unchanged renal excretion rate of radio-metabolites after pre-treatment with α-CCA-Na in the piglets.

Examination of the PET scans of the control piglets (Figure 5B,C) indicated some progressive uptake in the vertebrae, most likely due to defluorination of [^18^F]FACH.

### 2.6. Metabolism of [^18^F]FACH in Mice and Piglets In Vivo

**Figure 6:**
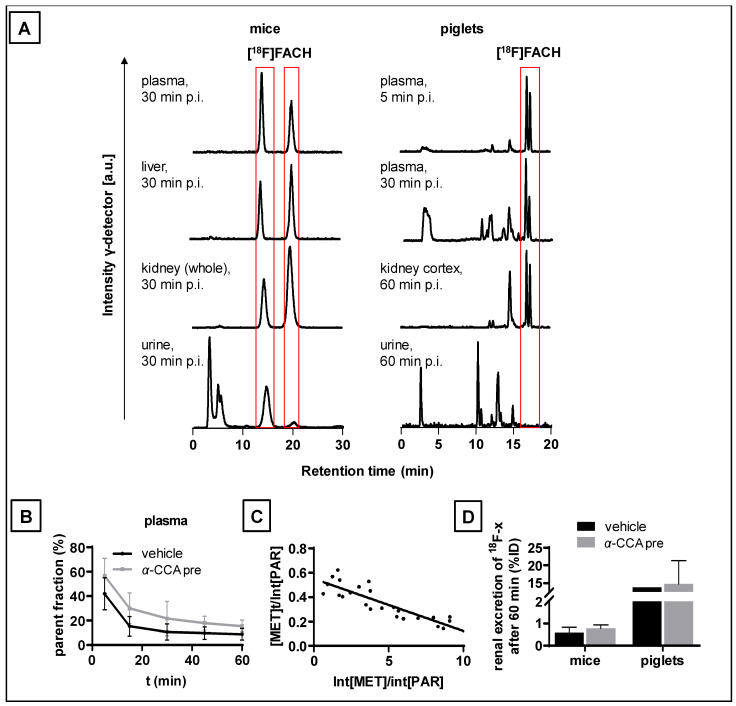
Metabolism and renal excretion of [^18^F]FACH in mice and piglets. (**A**) Representative radio-HPLC chromatograms of indicated tissues extracts (a.u.-arbitrary unit), red boxes indicating the neutral and deprotonated form (parent) of [^18^F]FACH [28]. (**B**) Parent radiotracer fraction over time in plasma of piglets in control (*n* = 3) and *α*-CCA-Na pre-treated group (*n* = 3). (**C**) Plasma metabolite plot for [^18^F]FACH in a representative piglet, where the ordinate intercept corresponds to the whole body fractional rate constant for [^18^F]FACH (*k*_0_, min^−1^), and the inverse of the linear regression slope indicates the fractional rate constant for elimination of the pooled plasma radio-metabolites (*k*_−1_, min^−1^) [32]. (**D**) Renally excreted ^18^F-activity as percentage of injected dose (%ID) after 60 min in piglets (control: *n* = 1, *α*-CCA-Na pre: *n* = 2) and mice (vehicle: *n* = 10, *α*-CCA-Na pre: *n* = 5; mean ± SD).

Representative radio- and UV-HPLC chromatograms for each analyzed tissue are shown in Figures S3–S11. More than 99% radioactivity was intact [^18^F]FACH in the mouse plasma samples at 30 min p.i. (Figure 6A). A neutral and a deprotonated form of [^18^F]FACH was detectable in the radio-HPLC chromatograms as reported previously [28]. We found low renal excretion of radioactivity in mice, mainly consisting of polar radio-metabolites and traces of [^18^F]FACH (<5%) in urine samples of the control (0.51 ± 0.39 %ID) and α-CCA-Na pre-treated group (0.90 ± 0.41 %ID) (Figure 6A,D).

In contrast to mice, polar radio-metabolites of [^18^F]FACH were abundantly present in the piglet plasma samples at early time points p.i. (Figure 6A,B). The parent radiotracer fractions decreased from 42 ± 13% at five min to 8.8 ± 4.8% at 60 min in the control piglets, and from 57 ± 14% to 16 ± 5% in the piglets pre-treated with α-CCA-Na at five min p.i. (Figure 6B). The plasma radio-metabolite plot in a representative piglet is shown in Figure 6C. The resultant mean apparent rate constant (*k*_0_) for [^18^F]FACH metabolism was 0.87 ± 0.34 min^−1^ in the control and 0.39 ± 0.14 min^−1^ in the pre-treated groups of piglets (*p* = 0.084), indicating very rapid metabolism, and a non-significant inhibition by α-CCA-Na. The corresponding rate constants for elimination of the radio-metabolites from the plasma (*k*_−1_) were 0.059 ± 0.019 min^−1^ in the control group and 0.020 ± 0.003 min^−1^ in the α-CCA-Na group, suggesting a competitive blockade of the renal elimination of polar radio-metabolites. However, analysis of urine samples collected at 60 min p.i. (Figure 2D and Figure 6A) indicated similar renal elimination of the radio-metabolites in a control piglet (13.9 %ID) and in samples from two α-CCA-Na pre-treated animals (19.5 and 10.2 %ID), whereas no intact radiotracer was detectable. Additionally, in the kidney cortex of two control piglets (Figure 6A and Figure S2), 50.5 and 48.0% of parent fractions of [^18^F]FACH were detected at 60 min p.i., versus only 17.0% in the piglet pre-treated with α-CCA-Na (Figure S2). Radio-HPLC analysis confirmed the displaceable binding of [^18^F]FACH to the kidney cortex in piglets, and revealed the presence of radio-metabolites in the tissue (Figure 6 and Figures S2, S9 and S10).

### 2.7. Kinetic Modelling of Renal [^18^F]FACH Uptake in Mice and Piglets

Table 4 includes the results of 1-tissue-compartment kinetic modelling (1-TCM) of [^18^F]FACH in the kidney cortex and the liver of the control and pre-treated mouse groups (α-CCA-Na and reference compound). Table 5 shows the corresponding results for the control and α-CCA-Na pre-treated piglets. Representative curve fits for mouse and piglet tissues are shown in Figure S12. Initial analysis indicated over-specification of the 2-TCM (Table S1) and we therefore focused on the 1-TCM for reliable estimation of *K*_1_ (mL g^−1^ min^−1^, the tissue influx), *k*_2_ (min^−1^, tissue clearance rate constant) and the total distribution volume (*K*_1_/*k*_2_ = V_T_ in mL g^−1^). The BP_ND_ was estimated as the ratio of [V_T(control)_/V_T(pre-treatment)_] − 1.

In mice, the *K*_1_ of [^18^F]FACH to the kidney cortex was not significantly altered after pre-treatment with α-CCA-Na or the reference compound. However, the magnitude of *k*_2_ was increased two-fold and more than 30-fold upon pre-treatment with α-CCA-Na and the reference compound, respectively. The V_T_ of [^18^F]FACH in the kidney cortex declined two-fold upon pre-treatment with α-CCA-Na and more than 60-fold upon pre-treatment with the reference compound. As in the kidney cortex, *K*_1_ in the mouse liver was unaffected by pre-treatment with both compounds, but *k*_2_ was significantly increased seven-fold after pre-treatment with the reference compound. However, the V_T_ of [^18^F]FACH in the mouse liver dropped seven-fold after pre-treatment with the reference compound, but was unaffected by α-CCA-Na.

The 1-TCM fitting showed adequate agreement with the tissue curves measured in a representative piglet PET study (Figure 6C). The *K*_1_ of [^18^F]FACH in the kidney cortex declined significantly 2.1-fold, whereas the *k*_2_ increased 2.8-fold after pre-treatment with α-CCA-Na. The V_T_ of the radiotracer in the piglet kidney cortex declined six-fold upon pre-treatment with α-CCA-Na, which suggests a BP_ND_ of 6.2. In the piglet liver there was no significant impact of α-CCA-Na on the kinetic constants.

### 2.8. Cellular Uptake Studies of [^18^F]FACH in a Triple Negative Breast Cancer (TNBC) Cell Line of Mouse

**Figure 7:**
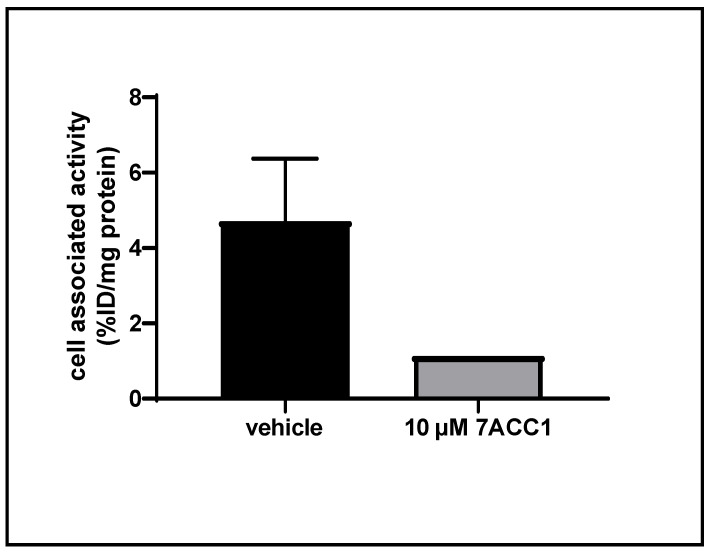
MCT1 specific cell uptake studies (37 °C, pH7.4, 5% CO_2_) of [^18^F]FACH in 4T1 cells. Either vehicle (*n* = 3, mean ± SD) or 10 µM 7ACC1 (*n* = 1) were added 10 min before addition of 200 kBq [^18^F]FACH. Results after a 30 min incubation period with the radiotracer are shown.

Preliminary cell uptake studies of [^18^F]FACH were performed to confirm the MCT1 specificity of [^18^F]FACH. In these studies, 4T1 cells were pre-incubated with or without the MCT1 specific inhibitor 7ACC1 (final concentration of 10 µM) before addition of [^18^F]FACH to the medium containing the cells. This provoked a distinct reduction of cell-associated radioactivity from 4.7 %ID/mg protein in control samples to 1.1 %ID/mg protein after 30 min incubation with the inhibitor (Figure 7).

### 2.9. Toxicity Studies of the Reference Compound in Rats

Toxicology studies in rats showed a no-observed-adverse-effect-level(NOAEL) of 620 µg FACH-Na/kg bodyweight, a dose more than 6200-fold higher than the estimated human dose based on the ICH guideline M3 (R2) and approximately 1000-fold the equivalent to the human dose (0.1 µg/kg), thus indicating a remarkably high margin of safety in PET studies.

## 3. Discussion

MCT1/4 are implicated in fundamental aspects of lactate shuttling in relation to normal physiology and in the pathophysiology of diseases such as cancer. In this regard, biomarkers revealing the metabolic adaption to alternative energy supplies such as lactate are of great interest not only for clinical research applications, but also for monitoring treatment strategies with MCT1 inhibitors (e.g., AR-C155858 and AZD3965) aiming to kill cancer cells by reduction of glycolysis and induction of intracellular acidification [3]. Therefore, we developed [^18^F]FACH to provide a non-invasive tool for molecular imaging of MCT1/4 in the living organism. Recently, we described the MCT1/4-specific inhibition of lactate uptake by FACH into rat brain endothelial and MDA-MB231 cells (IC_50_ = 11.0 nM and 6.5 nM, respectively), and reported on the radiosynthesis of [^18^F]FACH (Figure 1A,C), along with its dosimetry in piglets [28,29,31]. In the present study, we characterized [^18^F]FACH in some greater detail regarding its metabolism and biodistribution in healthy mice and piglets. We found high plasma protein binding, extensive and reversible uptake into the kidney cortex, as well as predominantly hepatobiliary excretion in both species (Figure 3, Figure 4, Figure 5 and Figure 6). However, we observed a profound species difference in the metabolic degradation of [^18^F]FACH, with rapid metabolism in piglets, but no biotransformation in CD-1 mice (Figure 6A–C).

We detected renal excretion of [^18^F]FACH radio-metabolites in both investigated species. Mouse urine contained only traces of [^18^F]FACH (<5%, Figure 6A), which is in accordance with the low *f*_p_ obtained from the protein plasma binding studies. Hence, the high plasma protein binding in vivo could at least explain the observed unspecific liver uptake in mice and piglets as shown in Figure 3B and Figure 5C [33,34]. We suppose that its high albumin binding diminishes the excretion of intact [^18^F]FACH via glomerular filtration, as recently described for [^68^Ga]EDTA and [^68^Ga]DTPA [35]. Indeed, other studies have shown that albumin binding reduces plasma clearance of the radiotracers in a species-dependent manner [36,37]. However, further studies are needed to clarify the three-fold higher renal excretion of [^18^F]FACH in the displacement studies compared to that in the control studies (Figure 4C). Assuming [^18^F]FACH is a substrate of MCTs, then it could be displaced intracellularly by *α*-CCA-Na or by the reference compound, thus explaining the transiently increased plasma activity concentration compared with the *α*-CCA-Na pre-treatment studies.

The in vitro binding studies indicated specific MCT tissue binding, with the highest *B_max_* of [^18^F]FACH present in the kidney cortex. The specificity of the binding to the MCTs was proven by pre-treatment with *α*-CCA-Na, which is a well-known inhibitor possessing 10-fold selectivity for the MCT1 compared to the other subtypes [30]. This in vitro result is consistent with the known expression of MCT1 at the basolateral side of the proximal tubule cells [12,38], and confirms the high and specific renal uptake that we observed in our PET studies. The binding of [^18^F]FACH to the blood vessels in cryosections of the mouse lung most likely depicts the MCT1 expression of bronchial epithelial cells [39]. Additionally, we confirmed the MCT1-specificity of [^18^F]FACH in a preliminary binding study using the murine 4T1 TNBC cell line, which expresses the MCT1 but not MCT4 (Figure 7) [16,40].

Although, MCTs are ubiquitously distributed in peripheral tissues, our ex vivo biodistribution and PET studies results in mice revealed a specific uptake of [^18^F]FACH only in the kidney (Figure 3, Table 1). We cannot presently exclude the possibility that [^18^F]FACH is also a substrate for MCTs, and thereby accumulates intracellularly. Since organs such as small intestine, liver, heart and blood cells also express the MCTs, we suppose that either there is limited transporter availability on the cell membrane in these tissues, or that the transport gradient of the MCTs directed from the intracellular to extracellular side may hinder the possible [^18^F]FACH uptake into these cell types under physiological conditions. However, significant uptake of [^18^F]FACH into the erythrocytes can be excluded, since the biodistribution studies revealed a red blood cell to plasma ratio <0.1. Notably, the MCT1-mediated import of lactate is essential for the production of glucose via gluconeogenesis in parenchymal liver cells and proximal convoluted tubule cells of the kidney [41], and likewise into muscle cells after high intensity exercise to improve energy availability and intracellular acid–base homeostasis [42]. In future studies we shall investigate the potential use of [^18^F]FACH to visualize such metabolic adaptions manifesting in reversed MCT1 transport direction. Other studies demonstrated disease-related alterations in the MCT1/4 expression, whereby the lactate exporter MCT4 was upregulated under hypoxic conditions, as was likewise MCT1, as necessary for metabolic micromilieu-dependent import or export of monocarboxylates into the cells [3,43]. Consequently, the MCTs play a crucial role in energy metabolism in various tissues [3,6,17], which calls for further studies to clarify the potential use of [^18^F]FACH PET in human diseases, including cancer. Regarding the liver, where the highly expressed MCT1 transports *L*-lactate into the parenchymal cells for gluconeogenesis [1], we observed no substantial displacement of [^18^F]FACH after pre-treatment with *α*-CCA-Na (Figure 5), in contrast to the partial blockade by the reference compound. Since [^18^F]FACH and its radio-metabolites are mainly excreted by the hepatobiliary route, we suppose that non-specific liver uptake of the radiotracer masks the MCT-specific uptake into this tissue.

Indeed, occupancy of [^18^F]FACH binding sites by the reference compound in the mouse kidney and liver could also explain the increased availability of the radiotracer in the blood pool, as shown by the two-fold higher plasma AUC_0–60min_ in the pre-treatment experiments compared to the control group (Figure 3B, Table 2). Furthermore, the displacement study revealed a 66% drop in the SUV after i.v. injection of *α*-CCA-Na (Figure 4B), which implies reversible tissue uptake of [^18^F]FACH in the kidney cortex. Nevertheless, further studies are needed to clarify the exact mechanism of the radiotracer uptake.

The PET studies in the piglets showed similar results to those in the mice with regard to the specific [^18^F]FACH uptake in the kidney and non-specific uptake into the liver. However, the rapid metabolism of [^18^F]FACH in the piglets led to lower availability of intact radiotracer in plasma and a decreasing TAC in the kidney (Figure 5). Although, the specific signal decreased to half due to the uptake of radio-metabolites in the kidney, however about 60% of this uptake could still be blocked by *α*-CCA-Na pre-treatment.

The 2-TCM (Table S1) proved to be over-specified for fitting the renal binding data in piglet and mice. Therefore, we focused our attention on the 1-TCM, which gave stable results. The magnitude of *K*_1_ in mice was unaffected by blocking agents, indicating that the initial [^18^F]FACH uptake in piglet tissues is unrelated to specific binding. However, the *α*-CCA-Na dose displaced around 50% of the specifically bound radiotracer in living piglets. Graphical analysis indicated rapid [^18^F]FACH metabolism (*k*_0_, min^−1^) and relatively slow net elimination of radio-metabolites (*k*_−1_, min^−1^), explaining the low percentage of intact [^18^F]FACH in the piglet plasma at late time points. Indeed, imprecision in the late phase of the arterial input function hinders the compartmental analysis in piglets, whereas compartmental analysis data from mice is favored by the near absence of radio-metabolites. The pre-treatment with *α*-CCA-Na substantially reduced the magnitude of *K*_1_ in the kidney cortex of piglets, but not in mice, which might reflect a species-dependent effect of the free fraction of the radiotracer. The [^18^F]FACH *V*_T_ in the piglet kidney cortex was approximately 25% higher than that in mice and the specific binding in the kidney cortex around five-fold higher in piglets. As presently implemented, the 1-TCM ignores the presence of [^18^F]FACH radio-metabolites in the tissues. Therefore, the unaccounted radioactivity to tissue HPLC analysis over the whole time course of the PET acquisition necessarily causes overestimation of the true V_T_ in piglets and makes it difficult to quantify the BP_ND_.

## 4. Materials and Methods

### 4.1. General

A more detailed description of all procedures is provided in supporting information (SI). [^18^F]FACH was produced with molar activities in the range of 65–330 GBq/µmol either by two-step or one-step procedures, as previously described [29]. *α*-CCA was purchased from Millipore Sigma (St. Louis, MO, USA), and *α*-CCA-Na and FACH-Na (reference compound) were prepared according to the previously described procedure [28,29].

### 4.2. Animals

Female adult CD-1 mice (36.0 ± 3.3 g) were obtained from the Medizinisch-Experimentelles Zentrum at Universität Leipzig (Leipzig, Germany) and female piglets aged 6 to 11 weeks and weighing 16.0 to 24.9 kg (dams: German Landrace x German Large White, sires: Piétran) were obtained from the Lehr- und Versuchsgut Oberholz (Großpösna, Germany).

### 4.3. In Vitro and Ex Vivo Plasma Protein Binding Studies

To measure plasma protein binding in vitro, a 1 mL portion of piglet plasma was incubated with 4.9 MBq [^18^F]FACH (10 µL) on a shaker (400 rpm) for 20 min at 37 °C. The plasma protein binding was also measured ex vivo in 10 µL portions of mouse plasma from retro-orbital blood samples collected 70 min after intravenous (i.v.) injection of 3.7 MBq [^18^F]FACH via a tail vein. The free and bound fractions of [^18^F]FACH were separated by centrifugation at room temperature (2000 *g* for ten minutes, Centrifree^®^ YM-30, Ultrafiltration Device; Merck KGaA, Darmstadt, Germany). Radioactivity was measured in aliquots of ultrafiltrate and in prefiltration plasma samples, using a Wizard 2470 γ-counter (PerkinElmer LAS, Rodgau, Germany), and the free fraction in the plasma (*f*_p_) calculated from the concentration ratio.

### 4.4. Autoradiographic Analysis of Radioligand Binding in Mouse Tissues In Vitro

Cryosections from the mouse kidney (10 µm) were cut on a microtome (Microm HM560, Thermo Scientific Microm, Fisher Scientific GmbH, Schwerte, Germany), mounted on glass slides (SuperFrost, Thermo Scientific Menzel, Fisher Scientific GmbH), dried for two hours at room temperature, and stored at −25 °C. Before the binding experiment, the tissue slices were dried under a stream of cold air, pre-incubated with PBS (pH 7.4) for 15 min at room temperature and dried again before incubation with PBS containing [^18^F]FACH (1.2 nM) without (total binding) and with different concentrations of FACH-Na (10 µM–0.1 nM; homologous competition) for 60 min. Specific binding to MCTs of [^18^F]FACH in cryosections of mouse kidney was determined after co-incubation of 1 nM [^18^F]FACH with vehicle or 300 nM *α*-CCa-Na or 300 nM reference compound (homologous competition).

The incubation was terminated by washing with ice-cold buffer (50 mM TRIS-HCl, pH 7.4) twice for two minutes followed by dipping in ice-cold demineralized water for 5 s and drying under a stream of cold air. The dried sections along with activity standards (1 µL aliquots of different dilutions of the incubation solution dried on microscopic slides) were exposed to phosphor imaging plates (FujiFilm Co., Tokyo, Japan) transferred after 120 min to a phosphor imager (HD-CR 35 Bio; Dürr NDT GmbH & Co. KG, Bietigheim-Bissingen, Germany). Regions of interest (ROIs) were drawn manually or automatically on the tissue slices or standard spots, and the pixelwise results converted to Bq/mg wet weight. Non-linear regression analysis was performed with GraphPad Prism (v.3.0 GraphPad Software, San Diego, CA, USA). *K_D_* and *B_max_* values were calculated by the simplified Cheng and Prusoff equation (http://www3.uah.es/farmamol/Public/GraphPad/radiolig.htm, accessed on 5 February 2021) [44]:

(1) $$K_{D}=K_{i}={\mathrm{IC}}_{50}-\mathrm{Radioligand}\;\left(M\right)$$

(2) $$B_{max}=\frac{\mathrm{Top}-\mathrm{Bottom}}{\mathrm{Radioligand}\;\left(M\right)/\left(K_{D}+\mathrm{Radioligand}\;\left(M\right)\right)}$$

Qualitative autoradiography was performed in cryosections of mouse lung (16 µm) obtained and processed by incubation with 1.2 nM [^18^F]FACH

### 4.5. Ex vivo Biodistribution Study

Adult mice (28 to 39 g bodyweight) under isoflurane anesthesia were pre-treated by i.v. injection of saline as control (*n* = 3 for each time point) or 25 mg/kg *α*-CCA-Na (*n* = 3 for each time point) ten min before i.v. injection of [^18^F]FACH (0.53 ± 0.06 MBq, 0.14 to 0.29 pmol/g bodyweight). The animals were euthanized by anesthesia overdose and cervical dislocation at five, 15 and 30 min after i.v. [^18^F]FACH administration, and the radioactivity concentrations in harvested tissues were measured by a gamma counter (PerkinElmer), decay-corrected and normalized to the administered dose and tissue weight, and expressed as SUV in their harvested tissues. The red blood cell to plasma ratio was calculated as described by Bower et al. [45]

### 4.6. PET/MRI Scans

The biodistribution of [^18^F]FACH in mice was assessed by small animal PET (Nanoscan, Mediso, Budapest, Hungary) with dynamic 60 min emission recordings, followed by T1 weighted GRE MR (TR = 15.0 ms, TE = 2.4 ms, FA = 25°) imaging for anatomical correlations and attenuation correction. The mice (*n* = 20) weighing 32.2 ± 2.9 g were anaesthetised with 2% isoflurane in 60% oxygen, and placed on a thermostatically heated animal bed. The animals then received i.v. injections of 0.9% saline (control), reference compound (10 mg/kg), or *α*-CCA-Na (25 mg/kg) ten min prior or 20 min after (displacement studies) [^18^F]FACH administration (2.3–8.6 MBq, 0.5–11.2 nmol/kg). List mode PET data were binned as a series of attenuation-corrected sinogram frames (12 × 10 s, 6 × 30 s, 5 × 60 s and 10 × 300 s) and were reconstructed by Ordered Subset Expectation Maximization (OSEM3D) with four iterations, six subsets and a voxel size of 0.4 mm³ (Nucline v2.01, Mediso).

The piglets (*n* = 6) were inititally anaesthetised with intramuscular injections of stresnil and ursotamin, and anaesthesia was maintained with intravenous ursotamin and midazolam, as required. The hematocrit was measured in an ear vein blood sample collected just prior to imaging. Either saline (*n* = 3) or 25 mg/kg *α*-CCA-Na (*n* = 3) in saline was administrated ten min before i.v. administration of [^18^F]FACH (261–327 MBq, corresponding to 218–852 pmol/kg) via an ear vein. Imaging was performed with a simultaneous hybrid PET/MR system (mMR Biograph; Siemens, Erlangen, Germany) equipped with dedicated phased-array surface. PET data were reconstructed into a 128 × 128 matrix (voxel size: 1.40 × 1.40 × 2.03 mm^3^) using the built-in OSEM3D algorithm with eight iterations, 21 subsets, and a 3-mm Gaussian filter. The attenuation and decay corrected dynamic sequence consisted of 32 frames as follows: 1 × 15, 11 × 30, 1 × 45, 5 × 60, 1 × 90, 5 × 120, 1 × 180, 4 × 240, 1 × 300, 2 × 360 s, whereas activity concentrations in VOIs are represented in units of SUV or percentage of injected dose (%/ID). After completing the dynamic PET recording, piglets were euthanized with an i.v. overdose of T61 (Intervet Deutschland GmbH, Unterschleißheim, Germany) and tissues were sampled for radio-metabolism studies in selected animals.

The analysis of reconstructed datasets was performed with PMOD software (v4.103, PMOD Technologies LLC, Zurich, Switzerland).

### 4.7. Metabolism Studies of [^18^F]FACH in Mice and Piglets

Radio-metabolites were analyzed by analytical and semi-preparative HPLC on a JASCO LC-2000 system (JASCO Labor- und Datentechnik, Gross-Umstadt, Germany), consisting of a PU-2080 Plus pump, AS-2055 Plus auto injector (100 μL sample loop) or a manual injection valve with a 5 mL sample loop, and a UV-2070 Plus detector coupled with a gamma radioactivity HPLC detector (Gabi Star, Raytest Isotopenmessgeräte GmbH, Straubenhardt, Germany). Data analysis was performed with the Galaxie chromatography software (Agilent Technologies, Santa Clara, CA, USA). Monitoring of UV absorption was done at wavelengths of 254 and 400 nm (400 nm was detected as λ_max_ for this class of compounds). For analytical and semi-preparative radio-HPLC analyses, Reprosil-Pur columns (C18-AQ 250 × 4.6 mm; 5 μm; and C18-AQ 150 × 4.6 mm; 10 μm; Dr. Maisch HPLC GmbH; Ammerbuch-Entringen, Germany) were used. Acetonitrile (ACN) mixed with a 20 mM aqueous solution of ammonium acetate (NH_4_OAc) was used as eluent.

[^18^F]FACH (33.8 ± 3.3 MBq in 200 μL isotonic saline) was injected in awake CD-1 mice (*n* = 4) via a tail vein. At 30 min after radiotracer administration, the mice were anaesthetised with isoflurane, and retro-orbital blood was sampled, followed by cervical dislocation and collection of released urine. In corresponding piglet studies (*n* = 5), plasma was separated from the arterial blood samples drawn during the PET recordings at certain time points.

In mouse studies, at 30 min after i.v. injection of the radiotracer [^18^F]FACH, the plasma was isolated by centrifugation of a blood sample at 10,000 rpm for 2 min. The plasma and urine samples were prepared for reversed phase HPLC (RP-HPLC) analyses as described in the following text.

Protein precipitation of plasma and tissue homogenates was performed by addition of ice-cold ACN/H_2_O (9:1, *v*/*v*) at a ratio of 4:1 (*v*/*v*) of the solvent mixture to each tissue sample (*n* = 2). The samples were vortexed for 2 min, stored on ice for 3 min, and the suspensions were centrifuged at 10,000 rpm at 4 °C for 5 min. For the second extraction, the precipitates were washed with 100 μL of the solvent mixture and subjected to the same procedure. The combined supernatants (total volume between 1 to2 mL) were concentrated at 70 °C under argon flow to a final volume of approximately 100 μL and analyzed by analytical radio-HPLC. The analyses were performed under isocratic conditions using 30% ACN/20 mM NH_4_OAc (aq., pH 6.8) at a flow rate of 1 mL/min. To determine the percentage of the radioactivity in the supernatants compared to the total radioactivity aliquots were taken at each step and, as well as the precipitates, quantified by gamma counting.

In piglet studies, similar procedures were implemented for preparing samples from plasma collected at 15, 30, 45 and 60 min p.i., with the exception of collecting the urine sample only at 60 min p.i. Additionally, in order to measure the radio-metabolites in the piglet kidney, the tissue was resected at 60 min p.i. and after washing in PBS, samples of the kidney cortex and medulla were isolated and homogenized in demineralized water (2 mL/g tissue) using a borosilicate glass mortar with ten strokes of a PTFE plunger at 1000 rpm (Potter S, Homogenizer, B. Braun Biotech, Sartorius AG, Göttingen, Germany). Protein precipitation was performed by addition of ice-cold ACN/H_2_O at a ratio of 4:1 (*v*/*v*) of organic solvent to each tissue sample. The samples were vortexed for 3 min, placed on ice for 3 min, and the suspensions were centrifuged at 12,000 rpm at 4 °C for 5 min. For the second extraction, the precipitates were washed with 200 μL of the solvent mixture and subjected to the same procedure. The combined supernatants (total volume between 68 mL) were concentrated at 70 °C under argon flow to a final volume of approximately 2 mL and analyzed by semi-preparative RP radio-HPLC. For comparison, a set of samples was also measured by the analytical RP radio-HPLC, resulting in comparable results of both methods. The analyses were performed in gradient mode using 10% ACN/20 mM NH_4_OAc (aq.) as eluent A and 90% ACN/20 mM NH_4_OAc (aq.) as eluent B under the following conditions:

Semi-preparative RP-HPLC: 0–5 min 100% A, 5–17 min up to 70% B, 17–18 min up to 100% B, 18–21 min 100% B, 21–22 min up to 100% A, 22–30 min 100% A, flow = 3.0 mL/min.

Analytical RP-HPLC: 0–5 min 100% A, 5–35 min up to 54% B, 35–36 min up to 100% B, 36–40 min 100% B, 40–41 min up to 100% A, 41–50 min 100% A, flow = 1.0 mL/min.

To determine the percentage of the radioactivity in the supernatants compared to the total radioactivity aliquots were taken at each step and, as well as the precipitates, quantified by gamma counting.

### 4.8. Kinetic Modelling

Kinetic analysis of reconstructed datasets was performed with PMOD software. First, image derived arterial input functions (IDIFs) were obtained from the left ventricle in mice and from an artery close to the kidney in piglet studies. IDIFs were corrected for partial volume effects, plasma fraction from hematocrit values (individually determined for each piglet directly before PET imaging; for mice set to 0.51 according to CD-1 Mouse Hematology sheet from Charles River Laboratories, 2011) and radio-metabolites (measured for piglets, and set to zero for mice). Tri-exponential metabolite-corrected fitted input functions were used for one and two tissue compartment modelling (1-TCM/2-TCM) of time activity curves (TACs) in tissue volumes of interest (VOIs). For both species the specific blood volume was set to 0.15 mL g^−1^ in the kidney cortex and 0.128 mL g^−1^ in the liver, according to published values [46].

By interpolation of the measured radio-metabolite fractions from the piglet PET studies, the continuous TACs for [^18^F]FACH and its radio-metabolites were calculated. Subsequently, the magnitudes of the whole body rate constant for [^18^F]FACH metabolism (*k*_0_) and the elimination rate constant for the radio-metabolites (*k*_−1_) under control and pre-treated conditions were calculated as described previously [32]. Graphical analysis of the integrals of the plasma time-concentration series of PET tracers and their radio-metabolite(s) follow a linear relationship [32,47], where the ordinate intercept is equal to the whole body fractional rate constant for the metabolism of the parent, designated *k*_0_ (min^−1^). The linear regression slope corresponds to the fractional rate constant for the elimination of the radio-metabolite from circulation, designated *k*_−1_ (min^−1^), which indicates the renal clearance of the radio-metabolite. By interpolation of the measured radio-metabolite fractions from the pig PET studies, we calculated the continuous time activity curves for [^18^F]FACH and its radio-metabolites, and then calculated the whole body metabolism rate constants *k_0_* and whole body clearance range constants *k*_−1_ in the control and pre-treatment conditions by graphical analysis.

### 4.9. Toxicity Studies of the Reference Compound in Rats

The extended single dose toxicity studies of FACH in male (*n* = 15) and female (*n* = 15) outbred Wistar rats were performed in the Biological Testing Laboratory (BTL) in Russia (Study Number 680/19). The test item FACH-Na was administered by single bolus i.v. injection at doses of 6.2, 62 and 620 μg/kg body weight (bw). Mortality, clinical pathology parameters (hematology and serum chemistry), organ weights and microscopic tissue parameters were investigated 24 h and two weeks after treatment.

### 4.10. Cell Uptake Studies

4T1 cells (kindly provided by István Krizbai’s group, Institute of Biophysics, BRC, Szeged, Hungary) were seeded at a concentration of 10^5^/well in a 24-well cell culture plate and then incubated for 6 h at 37 °C, 5% CO_2_ in 500 µL RPMI 1640 Media (Gibco, Thermo Fisher Scientific GmbH) supplemented with 10% FCS for adherence of the cells to the tissue culture plate. Afterwards, the cell culture media was replaced with 500 µL RPMI 1640 supplemented with 20 mM HEPES adjusted to pH 7.4 without FCS and incubated for 16 h at 37 °C and 5% CO_2_. 2 h before starting the cell uptake experiments, the incubation media was renewed (400 µL), and 50 µL of 100 µM 7ACC1 (AdooQ Bioscience, Irvine, CA, USA) or vehicle diluted in incubation media (1% DMSO) was added 10 min before adding 200 kBq of the radiotracer to a total volume of 500 µL per well. After a 30 min incubation, cells were collected on ice and washed three times with phosphate buffer saline and subsequently dispersed in 500 µL 0.1 N NaOH at room temperature for 10 min. The activity of 100 µL cell solution was measured in a gamma counter and normalized to initial dose. Protein content of 10 µL portions of cell solution was measured with a BCA Kit (Pierce, Thermo Fisher Scientific GmbH) relative to a BSA standard curve, and binding was normalized to units of %ID/ mg protein.

### 4.11. Statistics

Data are shown in mean ± standard deviation (SD). Group differences were tested by Student’s *t*-test, with *p* < 0.05 designated as significant. Area Under the Curves (AUCs) and corresponding 95% confidence intervals (CI_95%_s) were calculated with GraphPad Prism (v.8.2) following the assumptions described by Gagnon et al. [48].

## 5. Conclusions

[^18^F]FACH binds to MCTs in the kidney cortex in vitro and in vivo. We foresee that [^18^F]FACH PET could serve to study MCT-dependent transport mechanisms, and note that quantitation of [^18^F]FACH uptake in mouse PET studies is favored by the absence of radio-metabolites. The [^18^F]FACH metabolism should be validated in further species, especially in anticipation of human applications. The potential of [^18^F]FACH for imaging the MCT1/4 expression and function in different tumour entities should be investigated in future studies, since MCTs are important markers for the state of malignancy. Furthermore, dosimetry and toxicity studies indicate a large safety margin for the use of [^18^F]FACH in human PET studies.

## Figures and Tables

**Table 1:** Ex vivo biodistribution SUVs from mice at depicted time points.

Tissue	Control						*α*-CCA-Na		*p*-Value (30 min)
	5 min		15 min		30 min		30 min		
	Mean	SD	Mean	SD	Mean	SD	Mean	SD	
kidney	7.97	0.36	12.81	0.94	15.97	1.07	5.94	1.01	0.001
urine	0.82	0.19	4.32	1.30	4.57	3.99	11.30	6.84	0.108
liver	9.65	0.53	10.65	1.11	8.48	1.00	8.81	0.77	0.335
gall bladder	4.61	2.69	5.36	0.58	10.96	3.26	8.21	5.03	0.250
blood	1.85	0.29	0.91	0.20	0.82	0.05	0.95	0.06	0.021
plasma	3.77	0.57	1.76	0.45	1.69	0.17	1.92	0.07	0.044
brain	0.08	0.02	0.04	0.01	0.04	0.01	0.04	0.00	0.209
bladder	0.17	0.03	0.35	0.05	0.31	0.07	0.27	0.17	0.389
ovar	0.38	0.20	0.33	0.12	0.32	0.08	0.54	0.24	0.106
adipose tissue	0.12	0.07	0.18	0.10	0.15	0.05	0.11	0.03	0.144
spleen	0.37	0.04	0.21	0.07	0.22	0.06	0.28	0.06	0.161
pancreas	0.51	0.07	0.44	0.12	0.38	0.12	0.47	0.19	0.256
adrenal gland	0.80	0.33	0.94	0.31	0.51	0.16	0.61	0.18	0.242
stomach	0.16	0.02	0.13	0.03	0.20	0.03	0.27	0.10	0.215
lung	0.89	0.12	0.59	0.05	0.52	0.02	0.76	0.14	0.020
heart	0.74	0.05	0.53	0.10	0.58	0.02	0.76	0.10	0.016
femur	0.17	0.03	0.12	0.02	0.15	0.02	0.21	0.02	0.004
small intestine	0.56	0.11	0.66	0.06	1.11	0.13	1.29	0.34	0.225
caecum	0.18	0.03	0.15	0.02	0.19	0.05	0.27	0.06	0.054
large intestine	0.24	0.02	0.19	0.03	0.26	0.02	0.25	0.03	0.055

*p*-value: control vs. α-CCA-Na at 30 min p.i.

**Table 2:** Activity accumulation in mouse tissues (AUC_0–60min_) with and without pre-treatment with *α*-CCA-Na.

Tissue	Vehicle		*α*-CCA-Na		Reference Compound	
	AUC_0–60 min_ (SUV min)	CI_95%_	AUC_0–60 min_ (SUV min)	CI_95%_	AUC_0–60 min_ (SUV min)	CI_95%_
whole blood	59	54–64	63	57–69	125	113–136
kidney cortex	290	265–315	153	132–174	62	60–64
liver	450	417–483	417	379–456	250	227–273
gall bladder	481	360–602	486	349–624	1388	1156–1620
small intestine	99	67–131	180	105–255	405	316–494

**Table 3:** Activity accumulation in piglet tissues (AUC_0–60min_) with and without pre-treatment with *α*-CCA-Na.

Tissue	Vehicle		*α*-CCA-Na	
	AUC_0–60 min_ (SUV min)	CI_95%_	AUC_0–60 min_ (SUV min)	CI_95%_
plasma	36	33–38	40	35–44
kidney cortex	529	489–569	178	168–188
kidney medulla	2414	2022–2807	1264	956–1571
liver	317	293–340	286	273–299
gall bladder	1693	1168–2219	4453	3392–5513
vertebrae	88	74–102	100	105–144

**Table 4:** Compartmental analysis of PET recordings for [^18^F]FACH in mice pre-treated with vehicle (control) vs. with *α*-CCA-Na or the reference compound, V_T_ (1 − TCM) = *K*_1_/*k*_2_, BP_ND_ = (V_T(control)_/V_T(pre-treated)_) − 1. *p* < 0.05.

Tissue	Pre- Treatment	*K*_1_ (mL/g/min)	*p*-Value	*k*_2_ (1/min)	*p*-Value	V_T_ (mL/g)	*p*-Value	BP_ND_	AIC
kidney cortex	control	0.13 ± 0.06		0.02 ± 0.01		8.60 ± 4.55		1.29	–8.3 to 109.9
	*α*-CCA-Na	0.12 ± 0.06	0.321	0.04 ± 0.01	0.013	3.84 ± 2.59	0.038		
	reference compound	0.09 ± 0.04	0.120	0.66 ± 0.22	<0.001	0.14 ± 0.03	0.005		
liver	control	0.49 ± 0.24		0.08 ± 0.02		6.47 ± 2.49		0.04	11.1 to 88.3
	*α*-CCA-Na	0.53 ± 0.19	0.390	0.09 ± 0.01	0.172	6.21 ± 2.61	0.431		
	reference compound	0.49 ± 0.07	0.497	0.58 ± 0.16	<0.001	0.91 ± 0.38	0.002		

**Table 5:** Compartmental analysis of PET recordings for [^18^F]FACH in piglets pre-treated with vehicle (control) vs. with *α*-CCA-Na, V_T_ (1 − TCM) = *K*_1_/*k*_2_, BP_ND_ = (V_T(control)_/V_T(pre-treated)_) − 1.

Tissue	Pre- treatment	*K*_1_ (mL/g/min)	*p*-Value	*k*_2_ (1/min)	*p*-Value	V_T_ (mL/g)	*p*-Value	BP_ND_	AIC
kidney cortex	control	0.69 ± 0.11		0.06 ± 0.01		11.29 ± 1.47		5.2	9.5 to 155.7
	*α*-CCA-Na	0.29 ± 0.06	<0.001	0.17 ± 0.04	<0.001	1.82 ± 0.75	<0.001		
liver	control	0.64 ± 0.10		0.13 ± 0.05		5.12 ± 1.27		0.18	11.0 to 94.9
	*α*-CCA-Na	0.57 ± 0.14	0.283	0.17 ± 0.11	0.399	4.34 ± 2.30	0.323		

## Data Availability

The data presented in this study are available on request from the corresponding author.

## Supplementary Materials

The following are available online at https://www.mdpi.com/1422-0067/22/4/1645/s1, Figure S1: PET displacement study in gall bladder and small intestine of mice, Figure S2: Radio-HPLC analysis of [^18^F]FACH and its metabolites in kidney of piglets, Figures S3–S6: Radio- and UV-HPLC chromatograms of [^18^F]FACH and its metabolites in mouse plasma, kidney and urine, Figures S7–S11: Radio- and UV-HPLC chromatograms of [^18^F]FACH and its metabolites in plasma, kidney cortex and urine of piglets, Figure S12: Representative 1-TCM curve fits in kidney cortex and liver of mice and piglets, Table S1: 2-TCM analysis of kidney cortex of mice.
