# Supplementary material for: Preclinical Evaluation of [18F]FACH in Healthy Mice and Piglets: An 18F-Labeled Ligand for Imaging of Monocarboxylate Transporters with PET
Source: Int J Mol Sci. 2021 Feb 6;22(4):1645. doi: 10.3390/ijms22041645 (PMC7915902; doi:10.3390/ijms22041645)
Supplement: Supplementary file 1 [file ijms-22-01645-s001.pdf]

Supplementary information

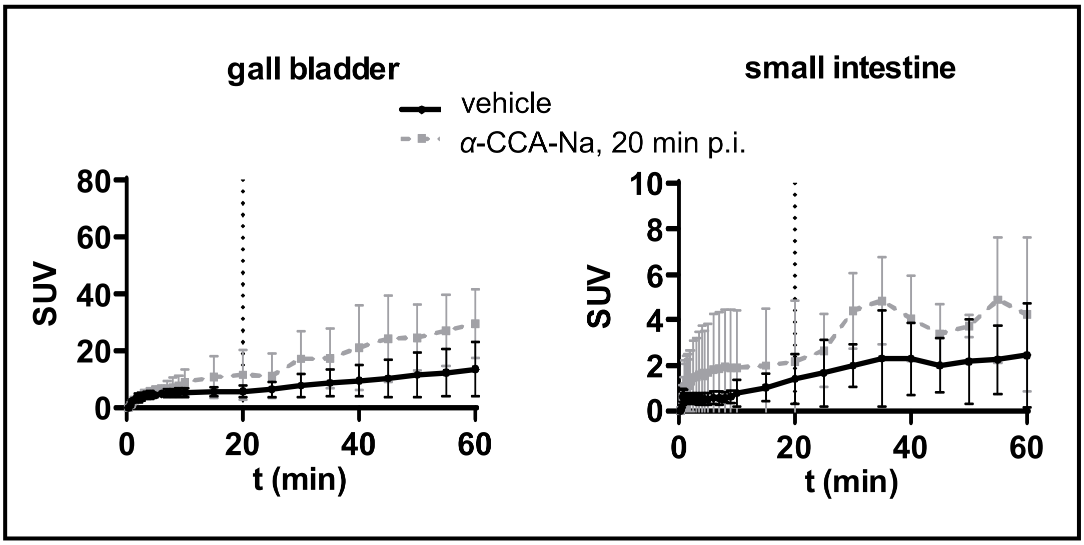

Figure S1. PET displacement study of [^{18}F]FACH in mice treated with α-CCA-Na (25 mg/kg) at 20 min after tracer administration (dotted line). Time activity curves of gall bladder and small intestine. Each point is the mean (±SD) of three determinations.

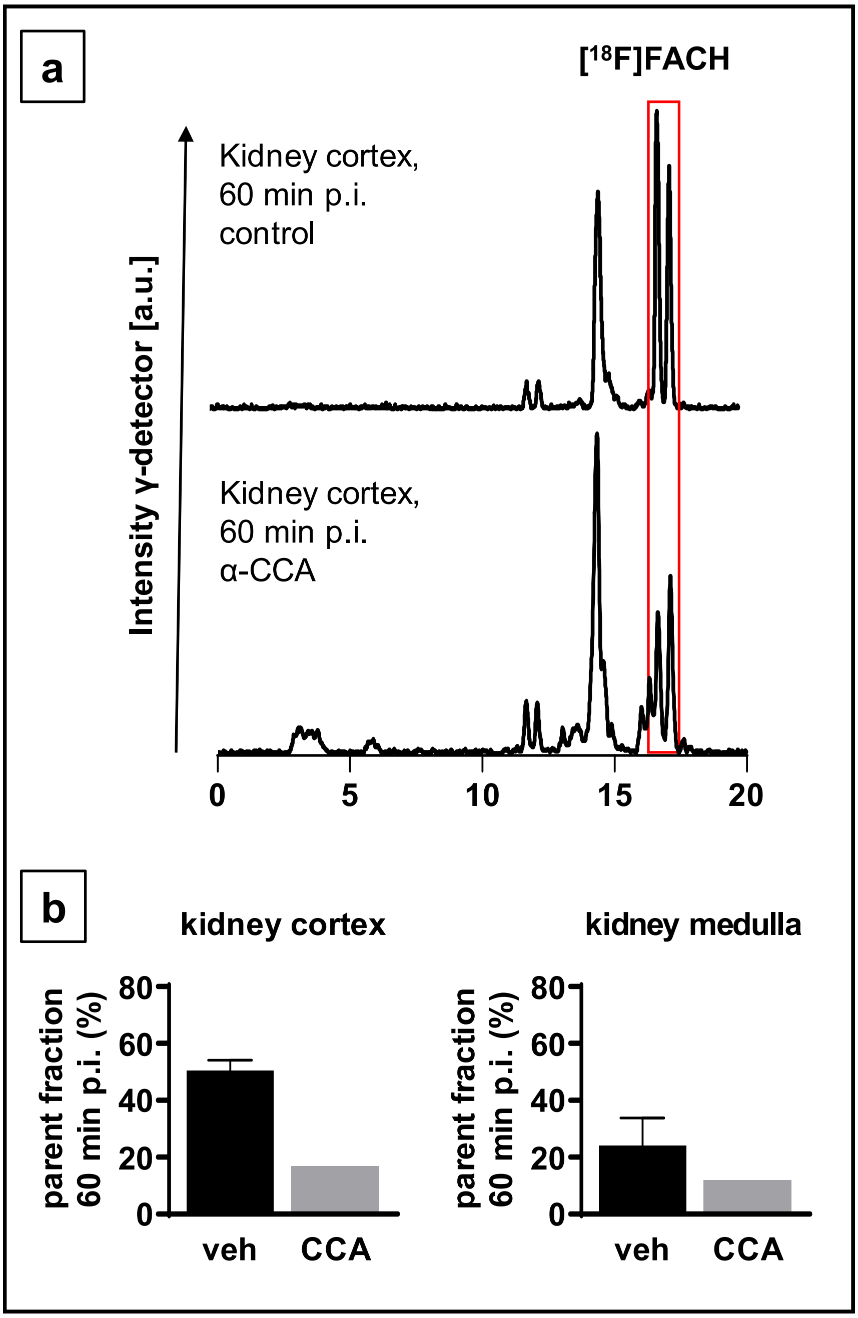

Figure S2 Radio-HPLC analysis of [^{18}F]FACH and [^{18}F]FACH-derived metabolites in the kidney of piglets pre-treated with (n=1) and without (n=2) α-CCA-Na subsequently sacrificed after PET imaging. a) representative radio-HPLC chromatograms (a.u. – arbitrary unit) of the kidney cortex, b) parent fraction determined in the kidney cortex and medulla.

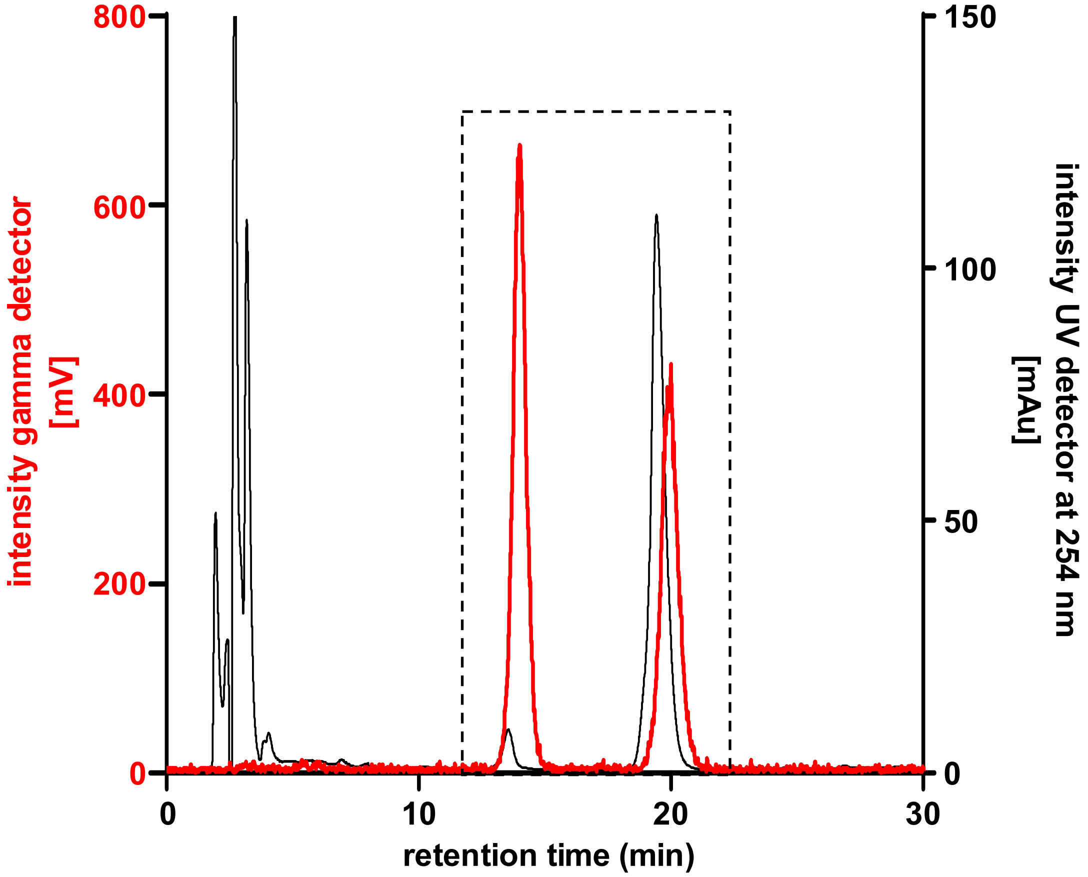

Figure S3 Representative radio (red line)- and UV (gray line)- HPLC chromatograms of extracted mouse plasma at 30 min p.i. of [^{18}F]FACH co-eluted with the reference compound, with parent fraction in the dotted square.

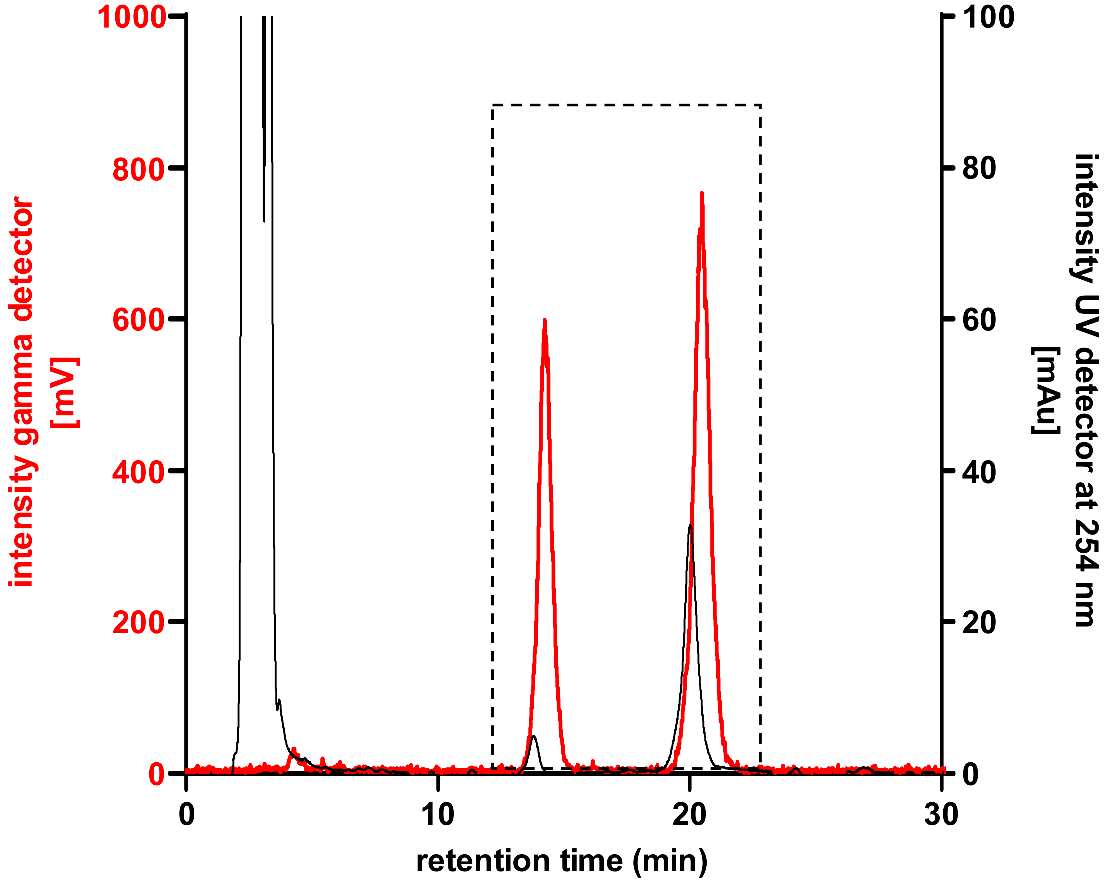

Figure S4 Representative radio (red line)- and UV (gray line)- HPLC chromatograms of extracted mouse liver at 30 min p.i. of [^{18}F]FACH co-eluted with the reference compound, with parent fraction in the dotted square.

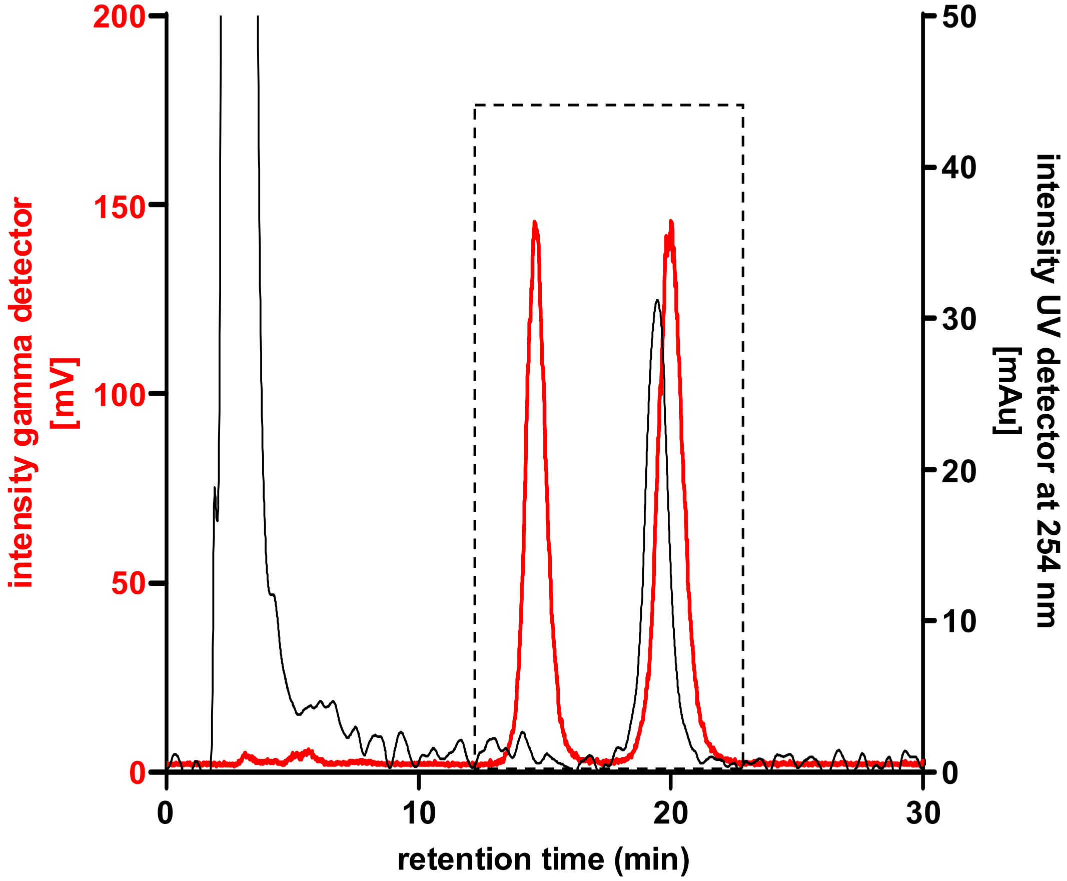

Figure S5 Representative radio (red line)- and UV (gray line)- HPLC chromatograms of extracted mouse kidney at 30 min p.i. of [^{18}F]FACH co-eluted with the reference compound, with parent fraction in the dotted square.

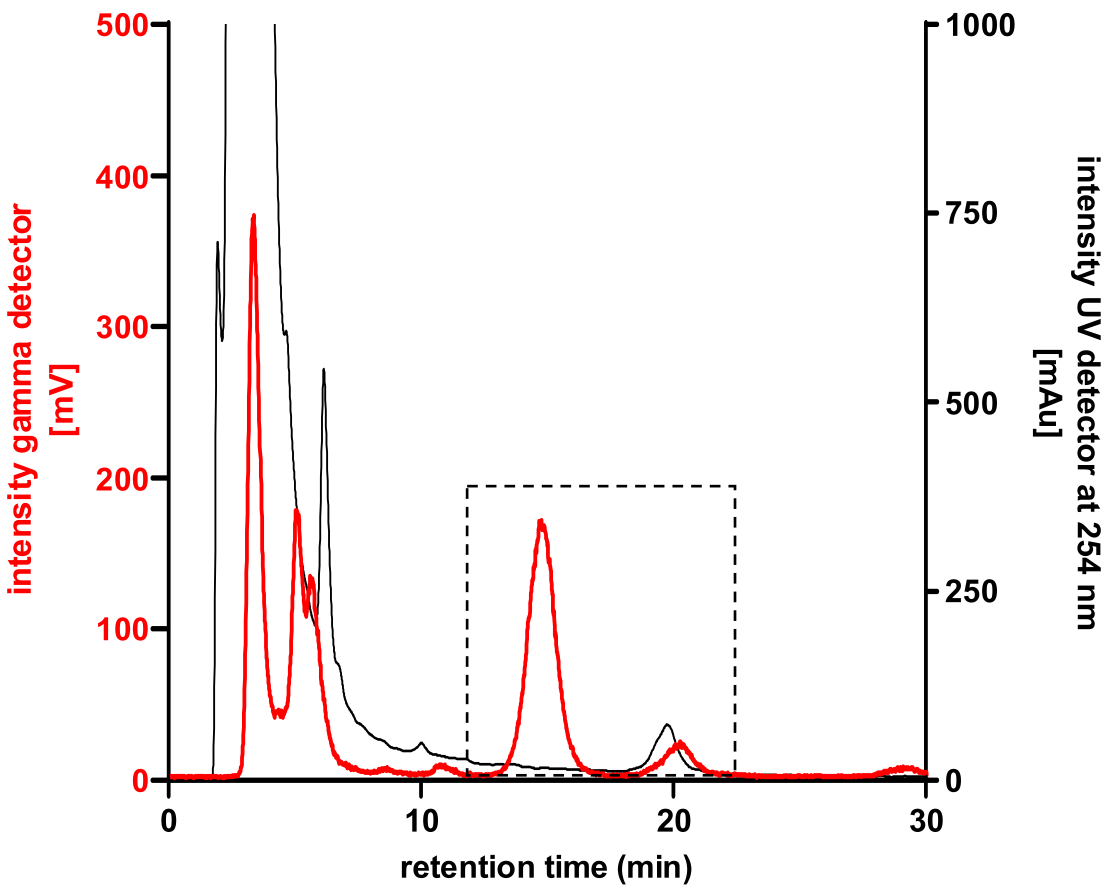

Figure S6 Representative radio (red line)- and UV (gray line)- HPLC chromatograms of extracted mouse urine at 30 min p.i. of [^{18}F]FACH co-eluted with the reference compound, with parent fraction in the dotted square.

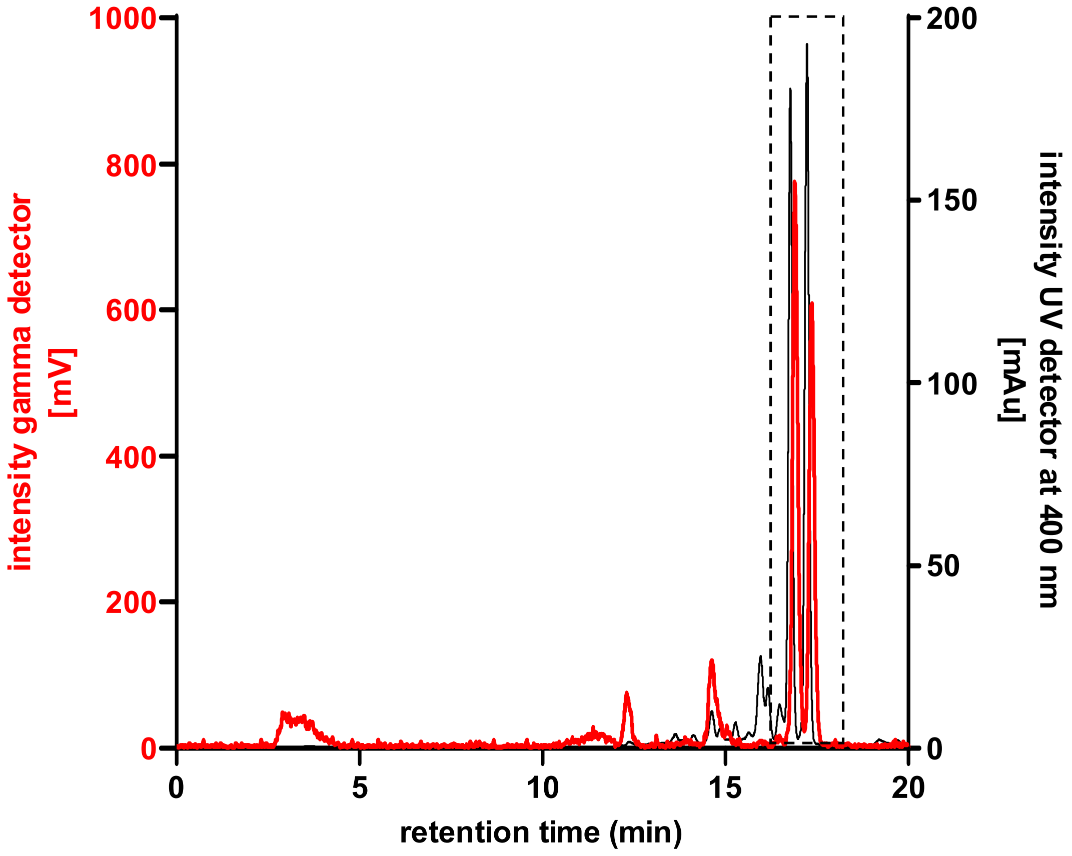

Figure S7 Representative radio (red line)- and UV (gray line)- HPLC chromatograms of extracted piglet plasma at 5 min p.i. of [^{18}F]FACH co-eluted with the reference compound, with parent fraction in the dotted square.

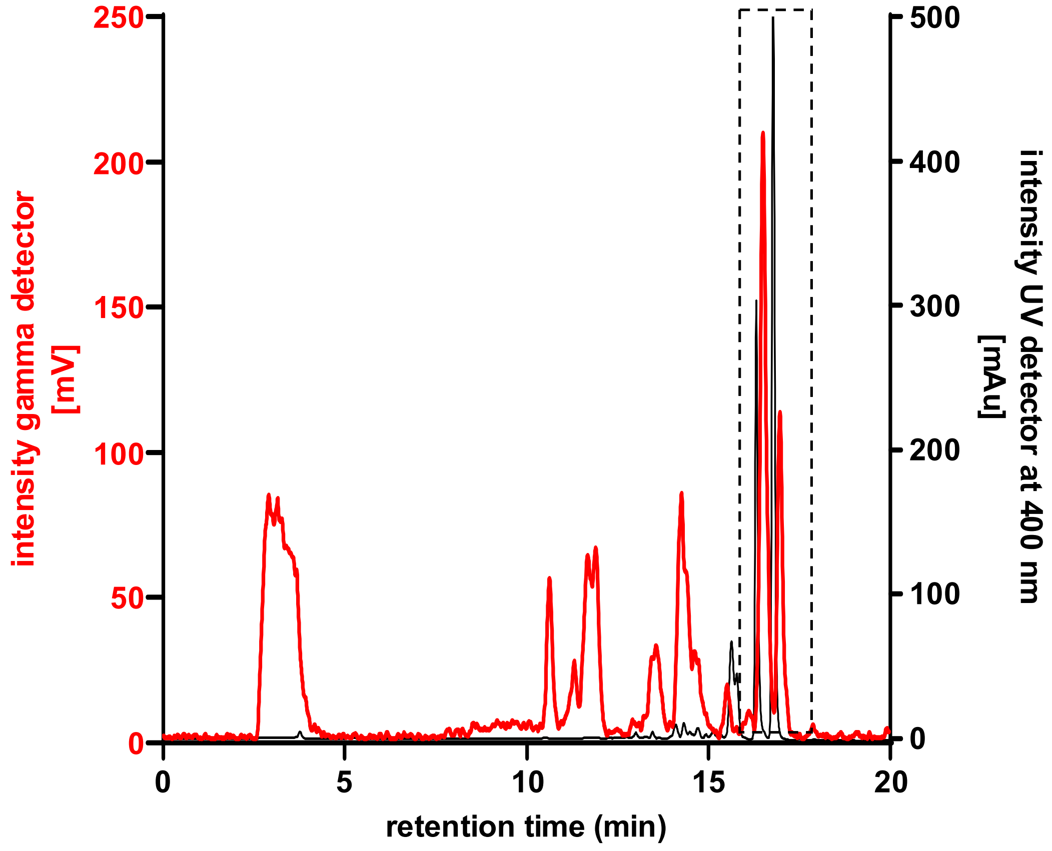

Figure S8 Representative radio (red line)- and UV (gray line)- HPLC chromatograms of extracted piglet plasma at 30 min p.i. of [^{18}F]FACH co-eluted with the reference compound, with parent fraction in the dotted square.

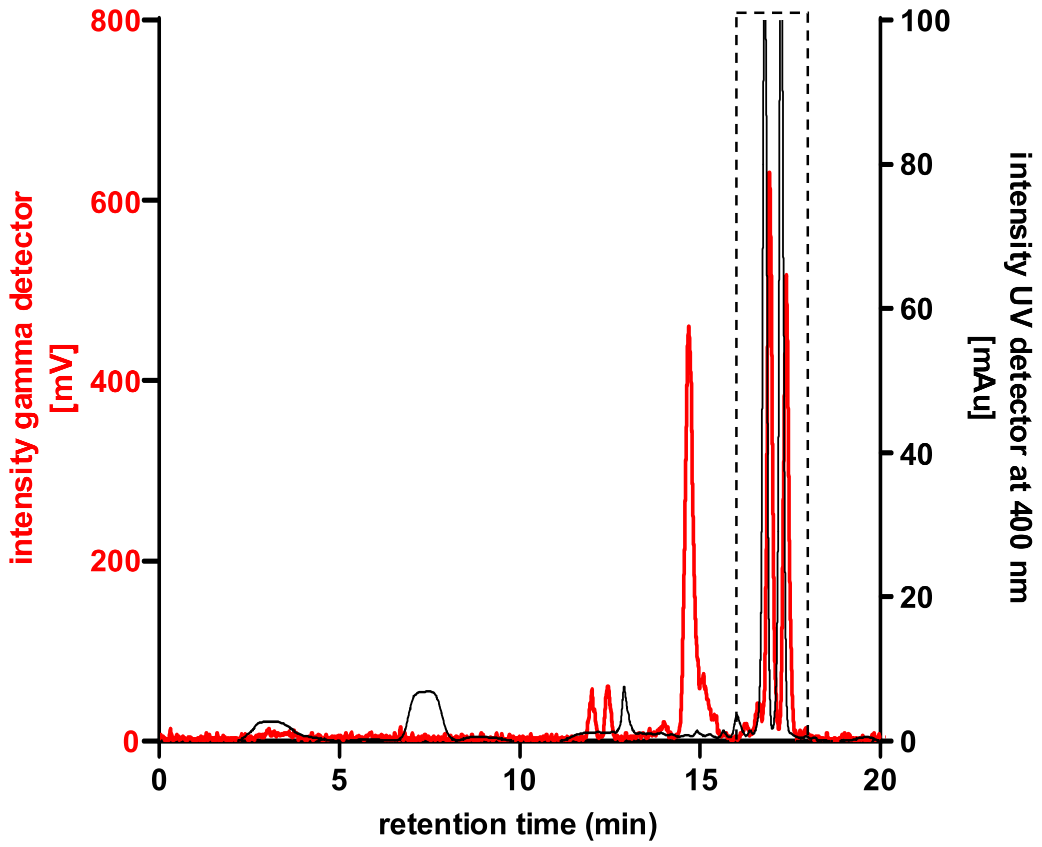

Figure S9 Representative radio (red line)- and UV (gray line)- HPLC chromatograms of extracted piglet kidney cortex at 60 min p.i. of [^{18}F]FACH co-eluted with the reference compound, with parent fraction in the dotted square.

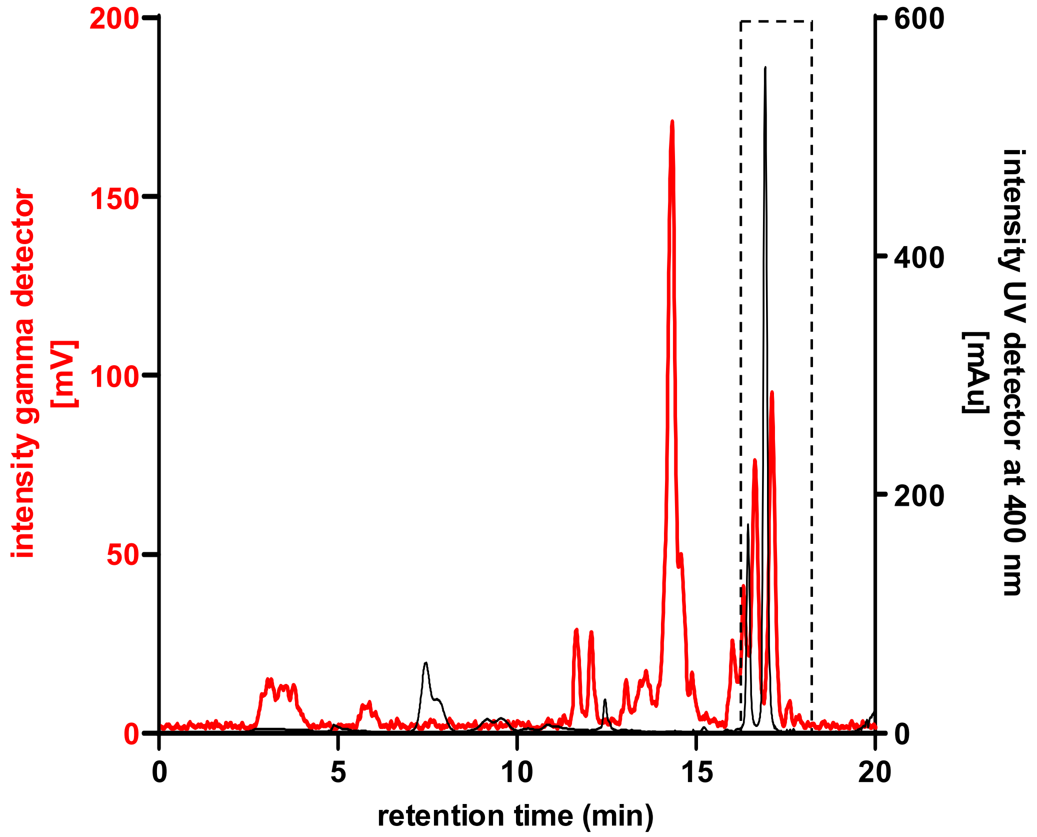

Figure S10 Representative radio (red line)- and UV (gray line)- HPLC chromatograms of extracted piglet kidney cortex at 60 min p.i. of [^{18}F]FACH pre-treated with α-CCA-Na co-eluted with the reference compound, with parent fraction in the dotted square.

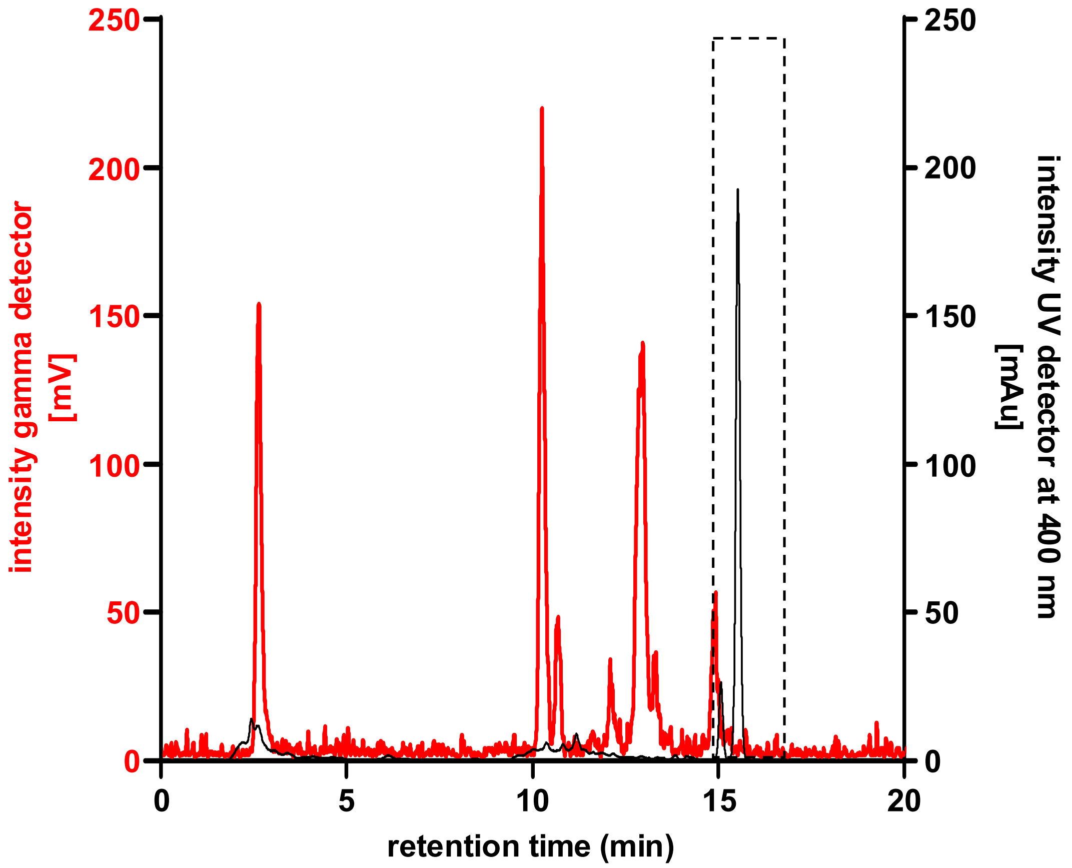

Figure S11 Representative radio (red line)- and UV (gray line)- HPLC chromatograms of extracted piglet urine at 60 min p.i. of [^{18}F]FACH co-eluted with the reference compound, with parent fraction in the dotted square.

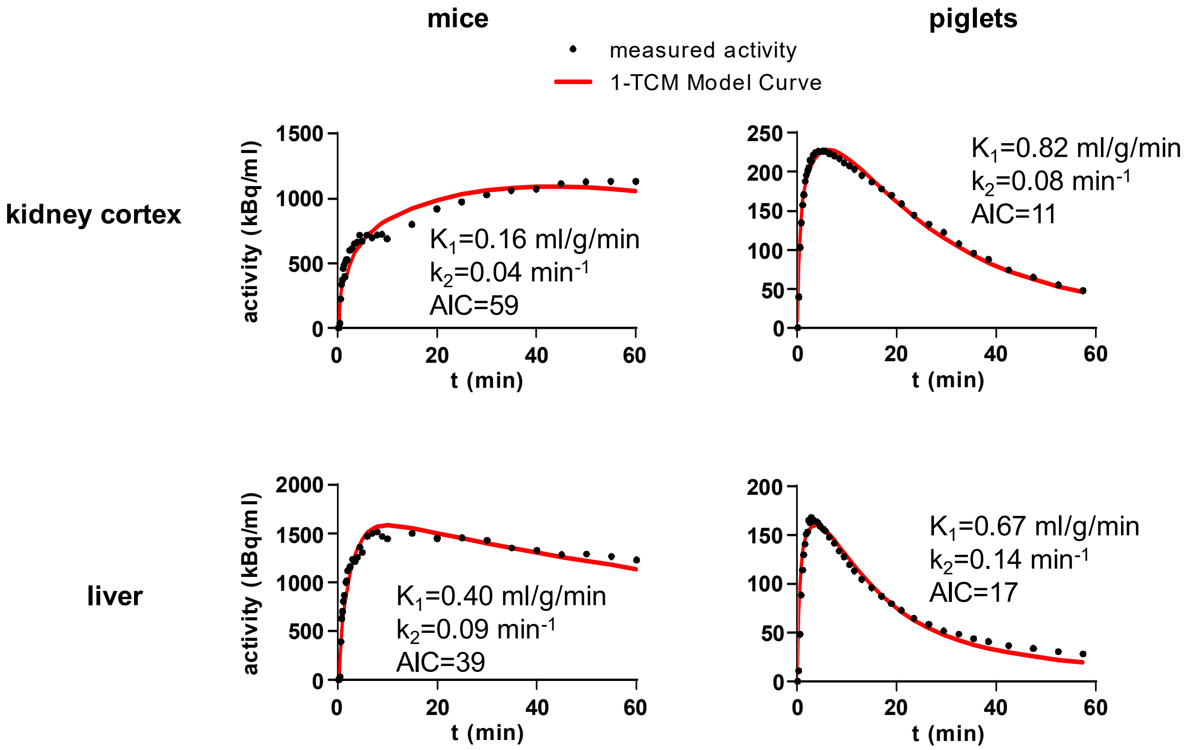

Figure S12 Representative 1-TCM curve fits of activity uptake over time into kidney cortex and liver of control studies in mice and piglets.

Table S1 2-TCM analysis of PET recordings for [^{18}F]FACH in kidney cortex of mice pre-treated with vehicle (control) vs. pre-treated with *α*-CCA-Na or FACH-Na, $V_T=K_1/k_2\ (1+k_3/k_4)$, $V_{ND}=K_1/k_2$, $BP_{ND}=k_3/k_4$.

pre-treatment	K_1 (ml/ccm/min)	k_2 (1/min)	V_T (ml/ccm)	V_{ND} (ml/ccm)	k_3 (1/min)	k_4 (1/min)	BP_{ND} (1/1)	AIC
Control	0.28±0.24	1.54±2.43	2.92×10^{294}	1.77±2.49	0.77±0.78	0.02±0.02	6.32×10^{293}	-8.3 to 109.9
α-CCA-Na	0.27±0.18	0.45±0.16	8.56±7.26	0.55±0.26	0.10±0.02	0.01±0.00	12.96±5.40	
FACH-Na	0.10±0.04	0.77±0.13	9.12×10^{102}	0.12±0.03	0.01±0.01	2.67±4.62	9.12×10^{103}	
